# Cross-kingdom RNAi: a universal mechanism of inter-organismal communication with many unknowns

**DOI:** 10.1093/jxb/eraf543

**Published:** 2025-12-18

**Authors:** Loukia M Kellari, Kalliope K Papadopoulou, Athanasios Dalakouras

**Affiliations:** Department of Biochemistry & Biotechnology, Laboratory of Plant and Environmental Biotechnology, University of Thessaly, Larissa, Greece; Department of Biochemistry & Biotechnology, Laboratory of Plant and Environmental Biotechnology, University of Thessaly, Larissa, Greece; Hellenic Agricultural Organization Demeter, Institute of Industrial and Forage Crops, Larissa, Greece

**Keywords:** Cross-kingdom RNAi, extracellular vesicles, host-induced gene silencing, microbe-induced gene silencing, small RNAs, spray-induced gene silencing

## Abstract

Cross-kingdom RNAi (ck-RNAi) is a biological process in which small RNA (sRNA) molecules are transferred between organisms belonging to different kingdoms to silence specific genes. Although numerous instances of reciprocal ck-RNAi have been documented in plants, demonstrating a modulation of the interaction between plants and their pathogens, pests, or symbiotic partners, the underlying molecular mechanisms remain largely elusive. In this review, we distinguish between naturally occurring and transgene-based cases of ck-RNAi, examine the diverse mechanisms governing the transfer of primary ck-RNAi signals from donor to recipient organisms, and explore the prerequisites for their amplification and systemic spread. Finally, we highlight key unresolved questions concerning the mechanistic basis of ck-RNAi and offer a perspective on its potential role in co-evolutionary dynamics.

## Teaser

This review summarizes current knowledge on transmission of small RNA between plants and their interacting organisms, highlighting the unexplored dimensions of the mechanism.

## Introduction

### The ever-moving small RNAs

RNA silencing, also known as RNA interference (RNAi) or post-transcriptional gene silencing (PTGS), is a conserved regulatory mechanism first observed in plants and later in fungi (‘quelling’) and animals (RNAi) (Napoli *et al.*, 1990; Fire *et al.*, 1991; Romano and Macino, 1992). It is initiated by dsRNA, which is processed by DICER-like (DCL) enzymes into small RNAs (sRNAs) that associate with ARGONAUTE (AGO) proteins to guide mRNA cleavage or translational inhibition (Agrawal *et al.*, 2003; Bartel, 2005; Fabian *et al.*, 2010; Jouravleva and Zamore, 2025). In plants, sRNAs can also direct DNA methylation through RNA-directed DNA methylation (RdDM) (Dalakouras and Wassenegger, 2013; Matzke and Mosher, 2014; Erdmann and Picard, 2020; Gallego-Bartolomé, 2020). Moreover, in plants, nematodes, and some fungi, RNA-DEPENDENT RNA POLYMERASEs (RDRs) amplify silencing via transitivity, generating secondary sRNAs from aberrant RNAs in a self-reinforcing cycle (Gazzani *et al.*, 2004; Wassenegger and Krczal, 2006; Baeg *et al.*, 2017; De Felippes and Waterhouse, 2020).

Conserved among all kingdoms are the two main classes of sRNAs, namely the siRNAs and miRNAs. siRNAs derive from perfectly complementary dsRNA precursors, originating either endogenously or exogenously from viruses or transposable elements, with a role in maintaining genome integrity and defense against viruses, transposons, and transgenes (Carthew and Sontheimer, 2009; Borges and Martienssen, 2015; Piombo *et al.*, 2024). Several variants of these siRNAs do exist in plants, each with its particular function and role: epigenetically activated siRNAs (ea-siRNAs), natural antisense siRNAs (nat-siRNAs), *trans*-acting siRNAs (ta-siRNAs), phased siRNAs (pha-siRNAs), pollen-siRNAs, tapetum-enriched siRNAs (tap-siRNAs), UV-induced siRNAs (uv-siRNAs), and virus-activated siRNAs (va-siRNAs) (Martínez De Alba *et al.*, 2013; Vaucheret and Voinnet, 2024). The second class of sRNAs, miRNAs, derive from endogenous genes, transcribed by RNA polymerase II to imperfect hairpin primary miRNAs, which are eventually processed by DCLs into mature 20–21 nt miRNAs that function as regulators in physiological processes, such as growth and development, immunity, and response to biotic and abiotic stresses (Mallory *et al.*, 2008; Voinnet, 2009). Besides these two canonical sRNA classes, non-canonical sRNAs that may not fulfill the criteria of bona fide siRNAs or miRNAs do exist. Perhaps one of the most important of these is tRNA-derived fragments (tRFs), encountered in both prokaryotes and eukaryotes; tRFs have a size of 17–40 nt, originate from nucleolytic cleavage (not necessarily by DICER) of mature or precursor tRNAs, and, among its other roles, can also associate with AGO and target transcript cleavage (Keam and Hutvagner, 2015; Alves and Nogueira, 2021).

**Fig. 1.**
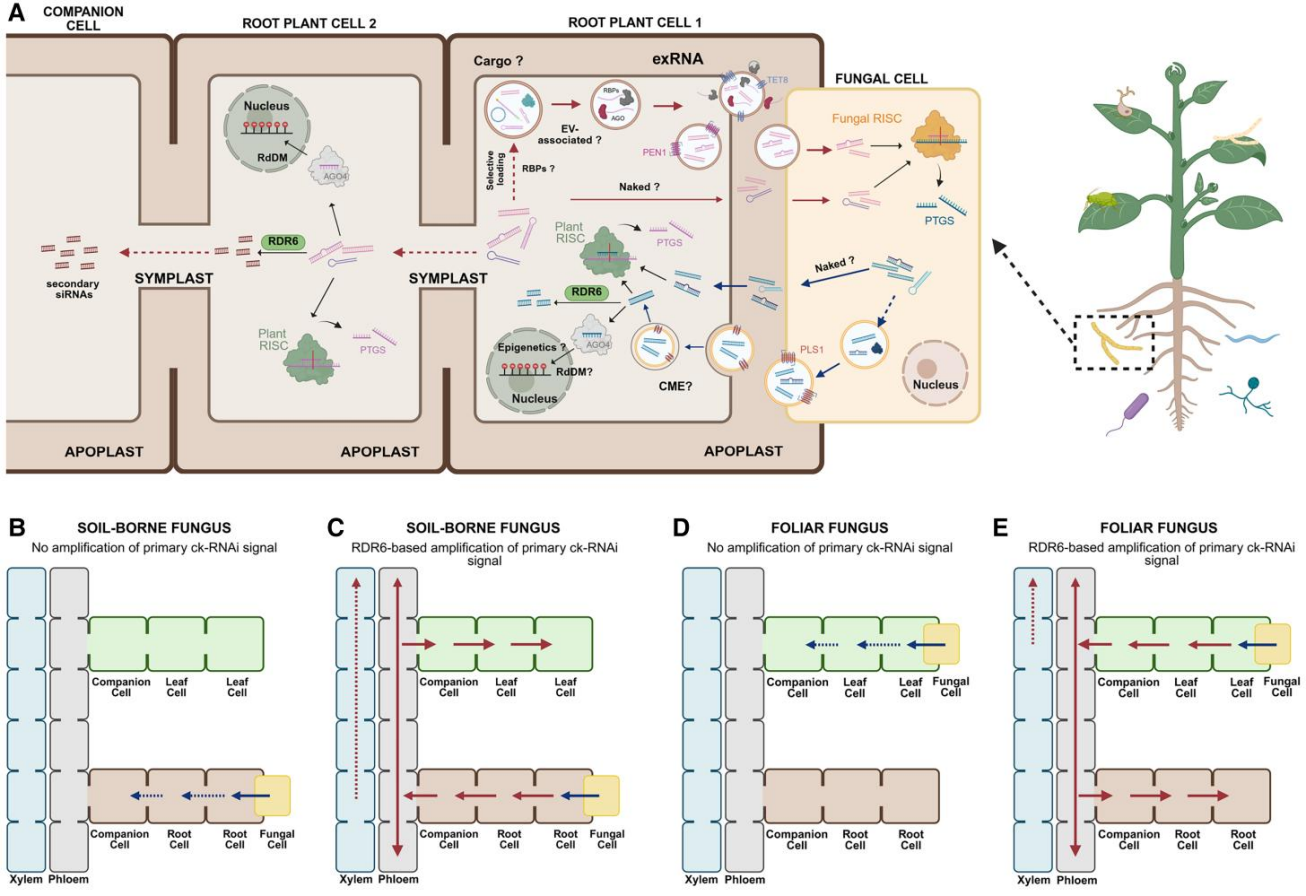
Summary of the molecular mechanisms underlying ck-RNAi. (A) Bi-directional sRNA translocation between aplant and a soil-borne fungus. Plant sRNAs (miRNAs, siRNAs, and tyRNAs) may be selectively packaged into PEN1/TET8-positive extracellular vesicles (EVs) (Cai *et al.*, 2018; Wang *et al.*, 2024; Koch *et al.*, 2025; Ravet *et al.*, 2025), potentially through the involvement of RNA-binding proteins (RBPs), or exist on the outer surface of EVs either freely or protein bound. They may also be transferred directly in ‘naked’ form to the fungal cells. Inside the fungus, these plant sRNAs are loaded onto the fungal AGO/RISC (RNA-induced silencing complex), triggering post-transcriptional gene silencing (PTGS) of corresponding fungal genes. Reversely, fungal sRNAs can be packaged inside PLS1-positive EVs (He *et al.*, 2023) and incorporated by the plant through clathrin-mediated endocytosis (CME) or even transferred in ‘naked’ form through a mechanism that is not clear. Inside the plant, the fungal sRNAs hijack the RNAi machinery of the host, triggering PTGS. Depending on their target, they may also recruit RNA-dependent RNA polymerase 6 (RDR6) which amplifies the primary sRNAs into secondary sRNAs. Primary or secondary sRNAs having a size of 24 nt may be loaded on host AGO4 and enter the nucleus to trigger RNA-directed DNA methylation (RdDM). Primary and secondary sRNAs can travel through the symplast (via plasmodesmata) to neighboring cells, triggering further PTGS, additional production of RDR6-dependent secondary sRNAs, and reinforcement of RdDM. While primary sRNAs will eventually be diluted away after moving through a certain number of cells, the ever-amplified secondary sRNAs can travel further and reach companion cells in the phloem, to eventually be systemically transported to distant plant parts. Created in BioRender. Kellari, L.M. (2025) https://BioRender.com/spyc57r. (B and C) sRNA translocation from soil-borne fungi to plant cells. Upon contact of the fungus with the plant root, fungal sRNAs are transferred into root cells. If the fungal sRNAs are not RDR6 amplified (blue arrows) (B), they can travel to a few neighboring root cells through the symplast, before their signal diminishes. In contrast, when the primary fungal sRNAs (blue arrows) are RDR6 amplified (C), the resulting secondary sRNAs (red arrows) can reach companion cells and travel through the phloem from source to sink; whether sRNAs can also be transported apoplastically and eventually move through the xylem to the upper parts of the plant is not clear. (D and E) sRNA transmission from foliar fungi to plant cells. Upon contact of the fungus with the plant leaves, fungal sRNAs are translocated into plant cells. If the fungal sRNAs are not RDR6 amplified (blue arrows) (D), they may travel to a few neighboring root cells through the symplast, before their signal diminishes. In contrast, when the primary fungal sRNAs (blue arrows) are RDR6 amplified (E), the resulting secondary sRNAs (red arrows) can reach companion cells and travel through the phloem; whether any apoplastic transportation takes place, is not clear. Created in BioRender. Kellari, L.M. (2025) https://BioRender.com/pfmbowp, https://BioRender.com/umkraom.

RNAi is not cell autonomous. Thus, in plants, sRNAs (both siRNAs and miRNAs) can move short distances to adjacent cells via the plasmodesmata or long distances via the vasculature to distant parts of the plant to establish systemic RNAi (Baulcombe, 2004; Melnyk *et al.*, 2011; Devers *et al.*, 2020; Voinnet, 2022, 2025). sRNA movement in plants follows the path of the photoassimilates in the ‘source-to-sink’ direction (Tournier *et al.*, 2006; Kobayashi and Zambryski, 2007). During cross-kingdom RNAi (ck-RNAi; Fig. 1Α), sRNAs are suggested to be transported via extracellular vesicles (EVs), and EV-associated sRNAs have been isolated from the apoplast, underpinning the notion that a mechanism of apoplastic sRNA communication cannot be excluded (Baldrich *et al.*, 2019; Voinnet, 2025). In fungi, intercellular transfer of sRNAs can be achieved through the septal pore, a plasmodesmata-like structure associated with endoplasmic reticulum (ER), or the desmotubule, a membranous cell wall-spanning structure that enables sRNA movement, either in naked form or enclosed in vesicles to move throughout the whole mycelial body (Wang and Dean, 2020). In nematodes, systemic RNAi requires the transmembrane proteins SYSTEMIC RNAi DEFICIENT (SID) (Winston *et al.*, 2002, 2007). Insects encode SID-like genes; coleopterans may even have two or three, but Dipterans seem to lack them altogether, perhaps accounting for the fact that the former are much more prone to RNAi than the latter. Yet, *Tribolium* exhibits systemic RNAi, which is independent of SID-like genes, suggesting that alternative systemic mechanisms do exist (Bucher *et al.*, 2002; Tomoyasu *et al.*, 2008). Systemic RNAi can also be observed in mammals, with sRNA movement being enabled via EVs, RNA-binding proteins, high-density lipoproteins (HDLs), and cell–cell structures, such as gap junctions or synapses (Soutschek *et al.*, 2004; Mittelbrunn and Sánchez-Madrid, 2012).

Breakthrough studies during recent years have demonstrated that sRNAs can be translocated not only inside an organism but also between organisms belonging to different kingdoms, in a phenomenon termed ck-RNAi (Weiberg *et al.*, 2013; Zeng *et al.*, 2019; Chen *et al.*, 2023; Mahanty *et al.*, 2023; Qiao *et al.*, 2023; Cheng *et al.*, 2025). Focusing on plants, naturally occurring ck-RNAi has emerged as a prominent mechanism of interspecies communication and has been observed during the molecular warfare between plants and their interacting pathogenic organisms (Weiberg *et al.*, 2013; Buck *et al.*, 2014; K. Zhu *et al.*, 2017; Cai *et al.*, 2018; Silvestri *et al.*, 2025). A clear distinction should be made between ck-RNAi and sRNA translocation within the plant kingdom, which has also been demonstrated between the parasite *Cuscuta campestris* and its host *Arabidopsis thaliana* (Shahid *et al.*, 2018), or even between two neighboring Arabidopsis plants (Betti *et al.*, 2021). In this review, we will focus only on ck-RNAi and discuss the most important cases of bi-directional ck-RNAi between plants and microbes and insects. Even before the discovery of natural ck-RNAi cases, artificial or transgene-based ck-RNAi methods were developed, mainly to be used as a powerful tool in crop protection platforms (Koch and Kogel, 2014; Koch and Wassenegger, 2021). Such approaches, including host-induced gene silencing (HIGS), spray-induced gene silencing (SIGS), and microbe-induced gene silencing (MIGS), are also briefly presented here. Further, we investigate the mechanisms that regulate the transfer of primary ck-RNAi signals between donor and recipient organisms, as well as the conditions required for their amplification and systemic dissemination (Fig. 1). Lastly, we emphasize the major open questions surrounding the mechanistic foundations of ck-RNAi.

## Cross-kingdom RNAi: natural cases

### Plants translocate sRNAs to microbes and insects

Plants transmit sRNAs to pathogenic microbes and pests as a natural defense strategy (Table 1). Diverse plant–fungal pathosystems have been reported: upon *Verticillium dahliae* infection, cotton plants increase the production of miR166 and miR159 and translocate them to the pathogen to silence genes essential for fungal virulence, namely a Ca^2+^-dependent cysteine protease (*Clp-1*) and an isotrichodermin C-15 hydroxylase (*HiC-15*) (Zhang *et al.*, 2016); upon *Botrytis cinerea* infection, *A. thaliana* transmits sRNAs that target genes related to vesicular trafficking, inhibiting pathogen infection (Cai *et al.*, 2018); upon *Fusarium graminearum* infection, wheat secretes miR1023 that silences the alpha/beta hydrolase gene of the pathogen, suppressing its invasion (Jiao and Peng, 2018); upon *Puccinia striiformis* infection, wheat translocates sRNAs to the pathogen to silence various genes (Mueth and Hulbert, 2022). Similarly, comparative analysis of transcriptome and sRNA expression patterns in the *Brachypodium distachyon*—*Magnaporthe oryzae* pathosystem predicted (but did not validate) the presence of several plant sRNAs silencing various fungal genes (Zanini *et al.*, 2021). Natural ck-RNAi was validated for the *Malus hupehensis*—*Botryosphaeria dothidea* pathosystem, where miR159a from apple silences the sugar transporter gene *BdSTP* of *B. dothidea*, affecting fungal growth and proliferation (Yu *et al.*, 2024). Recently, it was shown that upon *B. cinerea* infection, tomato sends sRNAs and silences serine/threonine kinase genes related to pathogenicity (Cheng *et al.*, 2025). Beyond fungal interactions, during the infection of Arabidopsis by the oomycete *Phytophthora capsici*, plant secondary siRNAs (siR1310) are translocated to silence *P. capsici* transcripts (Hou *et al.*, 2019).

**Table 1.** Summary of the reported studies about natural ck-RNAi occurring between plants and their associated organisms

From	To	Cargo	EV associated?*^a^*	Predicted or validated ck-RNAi*^b^*	Reference
From plants to microbes and insects					
*Arabidopsis thaliana*	*Botrytis cinerea*	miRNAs, siRNAs	Inside EVs (TET8+)	Validated	Cai *et al.* (2018)
*Arabidopsis thaliana*	*Phytophthora capsici*	siRNAs	EV-associated	Predicted	Hou *et al.* (2019)
*Arabidopsis thaliana*	*Botrytis cinerea*	mRNA	Inside EVs (TET8+)		Wang *et al.* (2024)
*Arabidopsis thaliana*	*Pseudomonas syringae*	Endogenous tyRNAs (10–17 nt)	Outside EVs (PEN1+, PEN3+) associated with RBPs	Predicted	Ravet *et al.* (2025)
			Inside EVs (TET8+)		
*Arabidopsis thaliana*	*Colletotrichum higginsianum*	sRNAs, long RNAs, AGO1, AGO2	Outside of high density EVs (PATL1+)	NA	Koch *et al.* (2025)
			Medium density EVs (TET8, RIN4, PATL1, ACTIN)		
			Low density EVs (RIN4, PATL1, PEN1, PEN3)		
*Arabidopsis thaliana*	*Plutella xylostella*	miRNAs	NA	Predicted	Zhang *et al.* (2019)
*Brachypodium distachyon*	*Magnaporthe oryzae*	sRNAs	NA	Predicted	Zanini *et al.* (2021)
*Gossypium* spp.	*Verticillium dahliae*	miRNAs	NA	Validated	Zhang *et al.* (2016)
*Malus hupehensis*	*Botryosphaeria dothidea*	miRNAs	EV-associated	Validated	Yu *et al.* (2024)
*Populus deltoides*, *Populus trichocarpa*	*Rhizophagus irregularis^c^*, *Laccaria bicolor^c^*	miRNAs	NA	Predicted	Mewalal *et al.* (2019)
*Solanum lycopersicum*	*Botrytis cinerea*	sRNAs	NA	Validated	Cheng *et al.* (2025)
*Triticum aestivum*	*Fusarium graminearum*	miRNAs	NA	Predicted	Jiao and Peng (2018)
*Triticum aestivum*	*Puccinia striiformis* f.sp. *tritici*	sRNAs	NA	Validated	Mueth and Hulbert (2022)
From microbes and insects to plants					
*Bemisia tabaci*	*Solanum lycopersicum*	sRNAs	NA	Predicted	Van Kleeff *et al.* (2016)
*Botrytis cinerea*	*Arabidopsis thaliana*, *Solanum lycopersicum*	sRNAs	NA	Validated	Weiberg *et al.* (2013)
*Botrytis cinerea*	*Arabidopsis thaliana*	sRNAs	NA	Validated	M. Wang *et al.* (2017)
*Botrytis cinerea*	*Arabidopsis thaliana*	sRNAs	Inside EVs (PLS1+)	Validated	He *et al.* (2023)
*Blumeria graminis* f.sp. *hordei*, *B. graminis* f.sp. *tritici*	*Hordeum vulgare*, *Triticum aestivum*	sRNAs	NA	Predicted	Kusch *et al.* (2018)
*Bradyrhizobium japonicum*	*Glycine max*	tRFs	NA	Validated	Ren *et al.* (2019)
*Hyaloperonospora arabidopsidis*	*Arabidopsis thaliana*	sRNAs	NA	Validated	Dunker *et al.* (2020)
*Fusarium oxysporum*	*Solanum lycopersicum*	miRNA	NA	Predicted	Ji *et al.* (2021)
*Pisolithus microcarpus^c^*	*Eucalyptus grandis*	miRNAs	NA	Predicted	Wong-Bajracharya *et al.* (2022)
*Puccinia striiformis*	*Triticum aestivum*	miRNA	NA	Predicted	B. Wang *et al.* (2017)
*Puccinia triticina*	*Triticum aestivum*	miRNA	NA	Validated	Dutta *et al.* (2019)
*Rhizophagus irregularis^c^*	*Medicago truncutula*	sRNAs	NA	Predicted	Silvestri *et al.* (2019)
*Rhizophagus irregularis^c^*	*Medicago truncutula*	sRNAs	NA	Validated	Silvestri *et al.* (2025)
*Sclerotinia sclerotiorum*	*Arabidopsis thaliana*	sRNAs	NA	Predicted	Derbyshire *et al.* (2019)
*Serendipita indica^c^*	*Brachypodium distachyon*	sRNAs	NA	Predicted	Šečić *et al.* (2021)
*Sinorhizobium fredii ^c^*	Soybean	Proteins, lipids	OMV-associated		Li *et al.* (2022)
*Trichoderma asperellum^c^*	*Solanum lycopersicum*	miRNAs	NA	Predicted	Y. Wang *et al.* (2021)
*Valsa mali*	*Malus domestica*	miRNAs	NA	Validated	Xu *et al.* (2022)
*Xanthomonas oryzae* pv. *oryzicola*	*Oryza sativa*	sRNAs	Inside OMVs	Validated	Wu *et al.* (2024)

^*a*^NA=not assessed.

^*b*^For validation, molecular methods are used to prove translocation of the sRNA to the recipient organism and cleavage of the mRNA target.

^*c*^Refers to beneficial non-pathogenic ck-RNAi cases.

So far, only a few cases of sRNA transmission from plants to beneficial microbes have been recorded. One example is the *in silico* prediction of the putative translocation of miRNAs from two *Populus* species (*Populus deltoides* and *P. trichocarpa*) to either an ectomycorrhizal fungus *Laccaria bicolor* or an arbuscular mycorrhizal fungus *Rhizophagus irregularis* during root colonization (Mewalal *et al.*, 2019). The predicted fungal gene targets of the *Populus*-derived miRNAs include transport proteins, transcription factors, and several genes encoding proteins of unknown function (Mewalal *et al.*, 2019).

For plant-to-microbe sRNA translocation to occur, it is not sufficient for the plant to produce and export sRNAs; the microbe must also be capable of perceiving these exogenous molecules. Indeed, the capacity to take up exogenous RNA is an essential requirement for a ck-RNAi event that not all fungi can manifest. For example, *Colletotrichum gloesporioides* is resilient to exogenous RNA uptake (Qiao *et al.*, 2021), in contrast to its close relative *C. truncatum* (Gu *et al.*, 2019). *Zymoseptoria tritici* is also resilient to exogenous RNA uptake; not surprisingly, a study investigating its interaction with wheat concluded that ck-RNAi was absent (Kettles *et al.*, 2019). However, fungal uptake of exogenous RNA must be distinguished from uptake of plant-derived RNA, as the latter can be delivered through EVs that facilitate sRNA internalization. Notably, even *C. gloeosporioides*, which is largely resistant to direct uptake of exogenous sRNAs, is susceptible to HIGS, indicating that EV-mediated delivery can overcome this barrier (Mahto *et al.*, 2020). An additional prerequisite for ck-RNAi seems to be the presence of the core RNAi machinery in the recipient organism. Most fungi, indeed, do encode RNAi genes (DCLs and AGOs), but these genes are not universally present across all fungal species. In a notable case, *Ustilago maydis* lacks the core RNAi machinery (no DCL, AGO, or RDR), in contrast to its close relative *U. hordei* (Laurie *et al.*, 2008). Yet, even bacteria, which lack canonical RNAi machinery, have been reported to exhibit susceptibility to HIGS (Ravet *et al.*, 2025). Whether this represents an isolated case or indicates a broader phenomenon remains unresolved.

Besides sending sRNAs to fungi and oomycetes, plants may also translocate sRNAs to bacteria and insects. *In silico* analysis predicts that Arabidopsis translocates several sRNAs, including tRFs, that are taken up by *Pseudomonas syringae* to silence bacterial genes (Ravet *et al.*, 2025). This was particularly striking, since bacteria were traditionally regarded as not being susceptible to RNAi. Yet, we should perhaps revisit this assumption, since in the same study it was shown that transgenic Arabidopsis plants expressing hairpin-derived sRNAs against *P. syringae* silenced the corresponding bacterial genes (see also below, ‘Host-induced gene silencing’) (Ravet *et al.*, 2025). Insects encode a well-studied RNAi pathway, and most species are susceptible to exogenous RNAs (Joga *et al.*, 2016; Dalakouras *et al.*, 2024). Plant miRNAs added in larval food regulated caste development of honeybees; when *Brassica campestris* miR162a was added in honeybee larval food, it targeted amTOR, a stimulatory gene in caste differentiation, preventing larval differentiation (K. Zhu *et al.*, 2017). Interestingly, plant miRNAs in larval food also affected *Drosophila melanogaster*, causing extended developmental times and reductions in body weight and length, ovary size, and fecundity (K. Zhu *et al.*, 2017). Arabidopsis miRNAs were reported to break the barrier of the insect mid-gut and enter the circulatory system of *Plutella xylostella* to silence hemocyanin domain-containing genes, eventually regulating insect development (Zhang *et al.*, 2019). Of note, an *in silico* study of sRNA sequencing data from the gut of the sup-feeding aphid *Myzus persicae* reveals the presence of 32 miRNAs in aphid gut samples, mapping not only to the genome of the host plant but also to predicted gene targets related to transcription of the aphid (Thompson *et al.*, 2019).

### Microbes and insects translocate sRNAs to plants

Ck-RNAi can be bi-directional, namely transmission of sRNAs can also occur from microbes to their plant hosts (Table 1). In a pivotal study, it was shown that *B. cinerea* sRNAs bind to Arabidopsis AGO1 protein and silence host immunity genes (Weiberg *et al.*, 2013). A few years later, a *Botrytis* sRNA effector (Bc-siR37) was identified that silences Arabidopsis defense-related genes (M. Wang *et al.*, 2017). In a similar fashion, the fungal pathogen *Puccinia striiformis* produces a novel miRNA-like (milR1) that suppresses the immune-related *PR2* gene during the interaction with wheat (B. Wang *et al.*, 2017). Moreover, *Puccinia triticina* translocates milR1, milR2, and milR3 to wheat to target host mitogen-activated protein (MAP) kinase, calmodulin, and F-box protein genes, respectively (Dutta *et al.*, 2019). Notably, a *Fusarium oxysporum* milR1 that is produced upon infection of *Solanum lycopersicum* was found to associate with plant AGO4a protein to regulate the expression of *SlyFGR4*, a gene implicated in defense against the pathogen (Ji *et al.*, 2021). The mechanistic details of sRNA uptake are not clear (see below), but a recent study showed that *B. cinerea* sRNAs ride in EVs to enter Arabidopsis cells through a clathrin-mediated endocytosis pathway (He *et al.*, 2023). Besides these cases, *in silico* studies have predicted that *Blumeria graminis* f.sp. *hordei* and *B. graminis* f.sp. *tritici* may send sRNAs to barley and wheat, respectively, to silence various genes (Kusch *et al.*, 2018). Similar predictions on sRNA translocation from pathogen to host have been made in the case of *Sclerotinia sclerotiorum* and Arabidopsis (Derbyshire *et al.*, 2019). sRNA translocation from fungal pathogens is not restricted to herbaceous species; *Valsa mali* milR1 and siR1 were shown to silence apple receptor-like kinase and disease resistance-related genes, respectively (Xu *et al.*, 2022; Liang *et al.*, 2024).

Research on sRNA transmission from symbionts to host plants is still limited, but is gradually expanding. The first example was an *in silico* prediction of sRNAs from *R. irregularis* with putative mRNA targets in the host *M. truncatula*, revealing a potential role during symbiosis (Silvestri *et al.*, 2019). Recently, the same research group validated that Rir2216 targets *M. truncatula* WRKY transcription factor 69 (MtWRKY69) to facilitate successful colonization (Silvestri *et al.*, 2025). Another mycorrhizal fungus, *Pisolithus microcarpus*, upon colonization of *Eucalyptus grandis* expresses novel miRNAs, including miR-8, which is transferred to *E. grandis* roots and silences NB-ARC domain-containing genes to establish symbiosis (Wong-Bajracharya *et al.*, 2022). *In silico* predictions have revealed probable ck-RNAi phenomena during the interaction of the root-colonizing endophyte *Serendipita indica* with *B. distachyon* (Šečić *et al.*, 2021) and miRNA-like molecules from *Trichoderma asperellum* were predicted to target tomato genes (Y. Wang *et al.*, 2021).

Not only fungi, but also oomycetes, bacteria, and insects translocate sRNAs to plants. Small RNA transmission has also been shown for the oomycete *Hyaloperonospora arabidopsidis*, which utilizes Arabidopsis AGO1 to silence host genes and thereby enhance its virulence (Dunker *et al.*, 2020). In prokaryotes, the nitrogen-fixing bacterium *Bradyrhizobium japonicum* produces tRFs that bind to the host AGO1 and silence host target genes during its interaction with *Glycine max* (Ren *et al.*, 2019). Moreover, *Xanthomonas oryzae* pv. *oryzicola* sRNA001 was shown to be loaded on outer membrane vesicles (OMVs) and transported to rice where it silenced the *JMT1* gene (Wu *et al.*, 2024), underpinning that our assumptions on the total absence of RNAi-like mechanisms in prokaryotes need urgent revision. Interestingly, insects also translocate sRNAs to their hosts; sRNAs from the whitefly *Bemisia tabaci* were detected in the phloem of tomatoes, probably transferred during feeding, although their gene targets were not experimentally validated (Van Kleeff *et al.*, 2016). Moreover, a recent study identified salivary miRNAs from the rice plant hopper *Nilaparvata lugens*, where miR-7-5P was deemed important for feeding and targets rice immunity genes (Zhang *et al.*, 2024).

## Cross-kingdom RNAi: artificial cases

### Host-induced gene silencing

Even before the documentation of natural sRNA translocation between organisms, RNAi-based technologies were employed in crop protection platforms. Such technologies include HIGS, which utilizes a transgenic plant (host) producing and delivering dsRNAs or sRNAs that target virulence genes to pests or pathogens. Excellent reviews on HIGS are available (Nowara *et al*., 2010; Koch and Kogel, 2014; Koch and Wassenegger, 2021; Niu *et al.*, 2021; Choudry *et al.*, 2024) and we will not dwell on details here. The most recent studies on HIGS in fungi include maize against *Aspergillus flavus* (Omolehin *et al.*, 2024), *Brassica napus* against *S. sclerotiorum* (Wytinck *et al.*, 2022), soybean against *F. oxysporum* (Pérez *et al.*, 2021), rice against *M. oryzae* (L. Zhu *et al.*, 2017), and wheat against *F. graminearum* (Wang *et al.*, 2020). In the case of oomycetes, HIGS has likewise been explored against *Bremia letucae*, which causes downy mildew in lettuce (Govindarajulu *et al.*, 2015), as well as *Phytophthora infestans*, a pathogen of potato (Jahan *et al.*, 2015). More recent studies have demonstrated successful HIGS against insect pests, including maize against *Chilo partellus* (Adeyinka *et al.*, 2023), cotton against *Aphis gossypii* (Zhang *et al.*, 2023), tomato against *Frankliniella occidentalis* (Venkatesh *et al.*, 2023), rice against *Chilo suppressalis* (Tang *et al.*, 2024), and potato against *M. persicae* (Murtaza *et al.*, 2022). Applications of HIGS extend further to nematodes—soybean lines expressing chitin synthase gene and tobacco plants expressing different chitin biosynthesis genes were rendered resistant against *Heterodera glycines* (Kong *et al.*, 2022) and *Meloidogyne incognita* (Mani *et al.*, 2020), respectively.

Apart from its importance as a plant protection method, HIGS has contributed to the elucidation of the molecular mechanisms that underlie ck-RNAi (reviewed in Koch and Wassenegger, 2021; Zand Karimi and Innes, 2022). For example, fungi preferentially take up long dsRNA molecules, with only some cases showing sRNA uptake (Koch and Kogel, 2014; Baulcombe, 2015; Zand Karimi and Innes, 2022). Herbivorous insects preferably take up long dsRNAs, which are then processed by their RNAi machinery (Koch and Wassenegger, 2021; Zand Karimi and Innes, 2022). In the case of sup-feeding insects such as aphids, HIGS mainly relies on sRNA rather than dsRNA uptake (Zand Karimi and Innes, 2022).

### Spray-induced gene silencing

An alternative to HIGS that relies on the exogenous application of dsRNAs or sRNAs to crops instead of using transgenic plants has been developed and termed SIGS (Dalakouras *et al.*, 2020; Chen *et al.*, 2023; Chen *et al*., 2025; Dubrovina *et al.*, 2025). In contrast to HIGS, SIGS seems to be a less efficient RNAi strategy since the sRNA/dsRNA application is transient, and repetition of application may be required. Yet SIGS can be more effective than HIGS in some cases, as has been shown in barley against *F. graminearum* (Koch *et al.*, 2019). Moreover, SIGS seems to gain ground due to concerns on GMO (genetically modified organism) use and the environment (Dalakouras *et al.*, 2024; Vatanparast *et al.*, 2024; Tardin-Coelho *et al.*, 2025). During SIGS, chemically synthesized sRNAs or *in vitro* and/or *in vivo* transcribed dsRNAs that target essential genes for survival and virulence in pests and pathogens are applied to plants. Methods of application include foliar spraying, trunk injection, or root drenching. Indicative SIGS reports include those against tobacco mosaic virus (Niehl *et al.*, 2018), potato spindle tuber viroid (Carbonell *et al.*, 2008), *F. graminearum* (Koch *et al.*, 2016), *P. infestans* (Kalyandurg *et al.*, 2021), *Leptinotarsa decemlineata* (Rodrigues *et al.*, 2021), *Tetranychus urticae* (Q. Wang *et al.*, 2025), and weeds such as *Amaranthus cruentus* (Hendrix *et al.*, 2021). A critical parameter for the efficacy of SIGS is the formulation of exogenous RNA, in order to increase its uptake from the target organism and protect it from environmental degradation; lipid double hydroxide (LDH) nanoclays and carbon dots are some of the most promising formulation agents for use in these cases (Mitter *et al.*, 2017; Schwartz *et al.*, 2020).

### Microbe-induced gene silencing

Upon the discovery of ck-RNAi between host and microbial pathogens, the interspecies RNAi (is-RNAi) between rhizospheric microorganisms was also investigated, resulting in the discovery of microbe-induced gene silencing (MIGS) (Wen *et al.*, 2023)—not to be confused with miRNA-induced gene silencing (also called MIGS) (Gauthier *et al.*, 2025). Compared with HIGS, MIGS does not require genetic manipulation of the host plant; compared with SIGS, MIGS does not rely on the use of nanomaterials to carry chemically synthesized RNAs. Still, MIGS is based on the use of a genetically modified microorganism, making it rather unsuitable for field application, at least under the current regulatory landscape. Wen *et al.* (2023), who first introduced the term MIGS, exploited the beneficial fungus *Trichoderma harzianum* to express dsRNA targeting the *O-mannosyltransferase* (*PMT*) gene, a well-known fungicide target, in *V. dahliae* and *F. oxysporum.* Growth inhibition of both pathogens was demonstrated in both *in vitro* and *in planta* experiments, with cotton plants showing protection against infection, when treated with the transformed *T. harzianum* strain (Wen *et al.*, 2023). Recently, MIGS was used to protect maize plants against *F. graminearum*, proving the applicability of the technology to various crops (Chen *et al.*, 2025). Another use of the MIGS-like case was shown by Porquier *et al.* (2021), where a *B. cinerea* strain lacking retrotransposon-derived sRNAs was complemented with the relative genes to reinstate their expression. This has led to enhanced infection and suppression of host defense-related genes, suggesting that retrotransposons are pathogenicity factors that manipulate host plant gene expression by *trans*-species sRNAs. In our own previous work, a variation of MIGS was implemented in a non-pathogenic, root-residing *Fusarium solani* strain K (FsK) fungal endophyte. Using a proof-of-concept system that consisted of an RNAi trigger [transgenic FsK expressing host green fluorescent protein (GFP)] and an RNAi-sensor (transgenic *Nicotiana benthamiana* expressing GFP), systemic silencing and DNA methylation of the host GFP was recorded upon *N. benthamiana* colonization with FsK (Dalakouras *et al.*, 2023; Kellari *et al.*, 2025).

## ck-RNAi in plant and non-plant systems

A common feature between ck-RNAi events in plant and animal systems is the delivery of microbial sRNAs aimed at modulating host immunity to promote infection (Weiberg *et al.*, 2013; B. Wang *et al.*, 2017). In mammals, *Heligmosomoides polygyrus* and *Heligmosomoides contortus*, two gastrointestinal nematodes, secrete miRNAs that suppress immunity in mice or murine and ovine organoids, respectively (Buck *et al.*, 2014; Perez *et al.*, 2025), but also during the interaction of a pathogenic bacterium suppressing immunity in human airway epithelial cells (Koeppen *et al.*, 2016). The same was also observed during sRNA transmission from the entomopathogenic fungus *Beauveria bassiana* to *Anopheles stephensi* (Cui *et al.*, 2022), reinforcing the conserved nature of this mechanism. In addition, hosts fight back and produce sRNAs to reduce the virulence of the parasites or pathogens, exemplified in this case by the transfer of sRNAs from *A. stephensi* to *B. bassiana* (Wang *et al.*, 2021). In an approach similar to MIGS in plants, a pathogen-mediated RNAi (pmRNAi) of *B. bassiana* transformed to express *Aedes aegypti* miRNAs (aaemiR-8 and aae-miR-375) acted as a negative regulator of the mosquito Toll immune pathway, thereby increasing fungal virulence (Cui *et al.*, 2022).

One probable common mechanism of sRNA trafficking between different organisms in both plant and animal interactions is through EVs (Fig. 1A). EVs are membrane-bound, phospholipid vesicular structures produced by both prokaryotic and eukaryotic cells, carrying nucleic acids, proteins, and lipids (Maas *et al.*, 2017; Sall and Flaviu, 2023). In recent years, their potential role in inter-organismal interactions has attracted research interest. During plant–pathogenic microbe interactions, plant EVs containing sRNAs (Cai *et al.*, 2018; He *et al.*, 2023; Ravet *et al.*, 2025) and even mRNAs (Wang *et al.*, 2024) were reported to be secreted. Bacteria can also produce OMVs, the bacterial analog of EVs, in pathogenic or mutualistic contexts. The phytopathogen *X. oryzae* pv. *oryzicola* produces the sRNA Xosr001d inside OMVs, which down-regulates rice immunity genes (Wu *et al.*, 2024), while *Sinorhizobium fredii* (now renamed *Ensifer fredii*) OMVs applied to soybean roots up-regulate nodulation and early symbiosis genes and down-regulate defense-related genes (Li *et al.*, 2022). In the animal kingdom, studies also suggest that sRNAs can move bi-directionally encapsulated in EVs from either the host or the pathogen. For example, in mammals, EVs were reported in the case of *Pseudomonas aeruginosa* and human airway epithelial cells (Koeppen *et al.*, 2016, 2021). EVs mediate the transfer of sRNAs between human monocyte cells and *Candida albicans* (Halder *et al.*, 2022). Nematodes are also capable of transferring EVs to their hosts, as observed in *D. melanogaster*, where they lead to down-regulation of antimicrobial peptide genes (Toubarro *et al.*, 2025), and in mouse cells, where they modulate host gene expression related to immunity and inflammation (Buck *et al.*, 2014).

In contrast to what happens with plant miRNAs, which directly cleave the target genes in pathogens (Zhang *et al.*, 2016; Cai *et al.*, 2018; Jiao and Peng, 2018), a study showed that human erythrocyte miRNAs, when taken up by *P. falciparum*, which lacks orthologs for AGO and DCL, inhibited mRNA translation by impairing their loading onto ribosomes (LaMonte *et al.*, 2012). A similar mechanism of non-canonical RNAi was also shown for *T. harzianum* sRNAs targeting *V. dahliae* through translational inhibition instead of mRNA cleavage (Wen *et al.*, 2023), although the exact mechanism was not uncovered. These examples illustrate that, although ck-RNAi is conserved across kingdoms, its mechanistic implementation varies widely, indicating the need for further investigation.

## Exploring the unknowns of ck-RNAi

Many key aspects of ck-RNAi in plants remain elusive, in particular as regards the movement of sRNAs between interacting organisms, their transport in distal parts of the host plant, as well as the factors determining their efficacy and specificity. Some of the current knowledge gaps that need to be addressed and clarified are summarized below.

### Are translocated sRNAs inside or outside EVs?

EVs not only protect RNA in the extracellular space where RNA and protein degradation enzymes are abundant, but also facilitate its transfer into destination cells (Hu *et al.*, 2025). Notwithstanding the recent developments in research on EVs and their role in sRNA translocation, the question of how the sRNAs are loaded inside EVs in the origin organism remains largely unresolved. In mammalian systems, several factors have been proposed, including sequence composition and structural features of the RNAs; involvement of RNA-binding proteins (RBPs); and different modifications either in the RNAs themselves or in the proteins they interact with, all of which could contribute to sorting (Dellar *et al.*, 2022). The selective loading of sRNAs inside EVs indicates that this process is also regulated in plants (Cai *et al.*, 2018; Baldrich *et al.*, 2019). It has been demonstrated that fungal sRNAs ride in EVs to enter plant cells through clathrin-mediated endocytosis (He *et al.*, 2023), although it is not clear whether clathrin-independent mechanisms also contribute in this process. However, the question is are the sRNAs inside or outside EVs. Whereas RBPs, like AGO1, RNA helicases, and annexins, can aid the transfer and/or stabilization of sRNAs inside EVs (Hagiwara *et al.*, 2015; He *et al.*, 2021), complexes of sRNAs with RBPs have also been found outside of EVs or in close proximity to the surface of EVs. Zand Karimi *et al.* (2022), showed that Arabidopsis apoplastic sRNAs are mainly located on the outside of EVs but associated with proteins that protect them against degradation. Similarly, Ravet *et al.* (2025), by applying various enzymatic treatments that degrade unprotected RNAs or proteins, reported that a significant titer of active sRNAs from apoplastic fluids are located on the outside of EVs (Fig. 1A). In a recent study in Arabidopsis, high-resolution density gradient ultracentrifugation classified the EVs into three categories based on their densities, noting that only the medium- and low-density fractions contain pure EVs associated with known markers, although sRNAs were found in the high-density fraction (Koch *et al.*, 2025). Since in most of the aforementioned studies EVs were isolated using different methods, resulting in the enrichment of different EV subpopulations, improving the accuracy of EV isolation methods will clearly minimize the risk of data misinterpretation. A sound assessment of the quality and purity of the EVs requires combined analysis of TEM, nanoparticle tracking analysis (NTA), and western blotting (Eldahshoury *et al.*, 2024). Intriguingly, it was recently found that various RNAs (tRNAs, rRNAs, mRNAs, miRNAs, and sRNAs), which are neither apoplastic nor in EVs or in protein complexes, are secreted directly onto the leaf surface rather than exuded through stomata or hydathodes (Borniego *et al.*, 2025), suggesting that diverse pathways of sRNA transport exist.

### Are sRNAs translocated as free molecules or AGO bound?

In plants, it is well demonstrated that 21, 22, and 24 nt sRNAs move systemically as AGO-free sRNA duplexes (Devers *et al.*, 2020) and, similarly during intraspecies RNAi between plants, miRNAs are translocated in AGO-free form (Betti *et al.*, 2021). During ck-RNAi between *Botrytis* and Arabidopsis, fungal sRNAs were reported to be translocated in AGO-free form (Weiberg *et al.*, 2013), while, upon uptake of the sRNA molecules, the use of the AGO proteins of the recipient organism seems to be a widespread phenomenon. This is true for tomato sRNAs binding to fungal AGOs (Cheng *et al.*, 2025), fungal (Weiberg *et al.*, 2013; Ji *et al.*, 2021) and oomycete sRNAs binding to plant AGOs (Dunker *et al.*, 2020), and even symbiotic bacterial sRNAs engaging with plant AGOs (Ren *et al.*, 2019). In animal systems, miRNAs from *B. bassiana* interact with mosquito AGO1, hijacking the RNAi machinery (Cui *et al.*, 2022). Thus, the overwhelming available experimental evidence suggests that sRNAs are translocated to plants as free molecules and are loaded on the host AGOs. In support of this argument comes the fact that during SIGS, the sprayed molecules are naked and not AGO-loaded sRNAs, but still exhibit biological activity once present in the host, indicating that they were eventually loaded on host AGOs.

### Onto which host AGO are the translocated sRNAs loaded?

Even if we assume that sRNAs are translocated as AGO-free molecules, it is not clear to which host AGO they will eventually be loaded. In plants, phylogenetic analyses have revealed three major clades of AGO proteins, which are named after Arabidopsis AGOs: AGO1/5/10, AGO2/3/7, and AGO4/6/8/9 (Fang and Qi, 2016). Thus, an AGO-free microbial sRNA, newly introduced into a plant cell, may be offered a variety of AGOs to be loaded onto. The sorting of sRNAs onto plant AGOs is primarily dictated by their size and the nucleotide at their 5′ terminus (Mi *et al.*, 2008). Hence, 21 nt sRNAs with 5′ U are loaded on AGO1 (the main mediator of PTGS), those with 5′ A on AGO2, and those with 5′ C on AGO5, while 24 nt sRNAs with 5′ A are loaded on AGO4/6/9 (the main mediators of RdDM). There is no reason to assume that exogenous/microbial sRNAs do not follow this biochemical rule once present in the plant environment; yet this hypothesis remains to be demonstrated and a comprehensive study analyzing their AGO association (e.g. with AGO-IP seq) and deciphering their eventual biological activity in the host is still lacking.

### Are all translocated sRNAs loaded on host AGOs?

In Arabidopsis, miRNAs differ in their AGO loading efficiency, and AGO abundance can be a limiting factor for miRNA incorporation (Dalmadi *et al.*, 2019). Similar constraints are likely to affect ck-RNAi: in cells where AGO levels are limited, not all translocated sRNAs may be loaded onto AGO, reducing ck-RNAi efficacy locally. However, sRNAs that fail to load may remain mobile and may still be incorporated into AGO complexes in adjacent cells, where they can initiate ck-RNAi. Consistent with this hypothesis, recent work in Arabidopsis indicates a negative relationship between AGO loading and mobility, with miRNAs remaining mobile precisely because they are not loaded (Fan *et al.*, 2022; Gonzalo *et al.*, 2025). Together, these observations suggest that non-cell-autonomous ck-RNAi may rely on the stepwise association of mobile sRNAs with AGO in recipient cells, ensuring a continuous pool of unbound sRNAs capable of further intercellular movement.

### What is the role of sRNA modification?

Which sRNAs are eventually translocated? All or some of them? It seems counterintuitive that sRNAs are indiscriminately loaded on EVs. Indeed, it has been suggested that in the Arabidopsis–*Botrytis* pathosystem, although the more abundant sRNAs are more likely to be transported through EVs, there is a clear selection in transferred sRNAs (Cai *et al.*, 2018). A molecular tag could be envisaged to somehow recruit those sRNAs that will preferentially be loaded on EVs over others. Interestingly, it has been recently shown that small and long RNAs associated with EVs from the leaf apoplast are enriched in *N*^6^-methyladenosine (m6A) modification (Zand Karimi and Innes, 2022), suggesting that such a type of modification might affect sRNA sorting on EVs. Whether additional RNA modifications also contribute to such a selection is not clear. In plants, HUA ENHANCER OF SILENCING 1 (HEN1) is an enzyme that modifies sRNAs by 2′-*O*-methylation, protecting sRNAs from degradation, particularly from 3′-uridylation, thereby increasing their stability and functional life span. Besides plants, HEN1 homologs that methylate sRNAs for protection have also been identified in mouse, zebrafish, and *Drosophila* (Ji and Chen, 2012). It is not clear whether fungal sRNAs contain this modification and/or whether this modification has any effect on sRNA translocation.

### Which types of RNA molecules are translocated?

A growing body of evidence suggests that sRNAs (miRNAs or siRNAs) of sizes typical of the organism of origin (20–24 nt) are the primary transferable molecules (Zhang *et al.*, 2016; Jiao and Peng, 2018; Hou *et al.*, 2019; Silvestri *et al.*, 2025). Notably, Baldrich *et al.* (2019) show that plant EVs are also enriched in small RNAs of 10–17 nt in length. These tinyRNAs (tyRNAs), as they were termed, represent degradation products originating from multiple sources, and their potential functional roles remain to be determined. Other reports showed the presence of mRNAs enclosed inside EVs of axenic cultures of *U. maydis* or Arabidopsis (Kwon *et al.*, 2021; Wang *et al.*, 2024), respectively, with the latter being incorporated by fungal cells and associated with polysomes for translation. Similar phenomena have been observed in mammalian systems, where mRNAs packaged within EVs are transported between different tissues and cell types (Valadi *et al.*, 2007; Yokoi *et al.*, 2017). Conversely, Zand Karimi *et al.* (2022), reported that Arabidopsis extracellular RNAs (exRNAs) did not contain mRNAs, but rather long non-coding RNAs (lncRNAs) and circular RNAs (circRNAs). Similarly in rice, circRNAs are involved in immune responses against the fungal pathogen *M. oryzae* (Fan *et al.*, 2020). Intriguingly, the question as to whether long dsRNAs (i.e. the precursors of sRNAs) are also translocated has not been addressed so far.

### What is the sequence complementarity prerequisite between the translocated sRNA and its target transcript in ck-RNAi?

It is reasonable to assume that, from a plethora of diverse sRNAs being translocated, only a few will exert meaningful biological activity on the recipient organism, namely only those which exhibit sufficient sequence complementarity with a host transcript. Perfect sequence complementarity will probably ensure maximum RNAi efficiency, but it is unclear how the number and occurrence of mismatches will affect ck-RNAi and to what extent. It is generally accepted that a perfect sequence complementarity of a 21 nt sRNA with its target transcript will be likely to result in mRNA degradation, while a single mismatch at position 10–11 may inhibit such cleavage (Iwakawa and Tomari, 2022). A few mismatches in other positions may not influence mRNA cleavage in some cases, but in others may lead to translational inhibition (Brodersen *et al.*, 2008). Translocated sRNAs having a size of 22 nt may also affect host gene expression by translational arrest (Franco-Zorrilla *et al.*, 2007; Wu *et al.*, 2020). Importantly, translocated sRNAs having a size of 24 nt may induce epigenetic modifications such as DNA methylation (Fig. 1A), even if the 24 nt sRNA contains up to four regularly interspaced mismatches compared with the target DNA (Fei *et al.*, 2021). All in all, how the necessary sequence complementarity in each case is maintained is still elusive; additional studies will be required to answer the question of whether it arises stochastically or through co-evolution of the host with the target organism (Knip *et al.*, 2014; Zhao and Guo, 2019; Nien *et al.*, 2024).

### Are the primary ck-RNAi signals sufficient for systemic function?

RDR6 seems to be involved not only in the generation but also in the systemic response to the ck-RNAi signal. Indeed, during ck-RNAi from the plant to a fungal pathogen, host RDR6 generates secondary sRNAs that constitute the primary host ck-RNAi signal to be translocated to the fungus. Thus, Arabidopsis *rdr6* mutants are more susceptible to *Phytophthora* infection, and *Phytophthora* even encodes a suppressor of silencing effector to inhibit secondary sRNA biogenesis in Arabidopsis and to promote infection (Hou *et al.*, 2019). Similarly, Arabidopsis *rdr6* mutants were more susceptible to *B. cinerea* infection, since Arabidopsis secondary sRNAs mediate the host defense against the pathogen (Cai *et al.*, 2018). On the other hand, we have also shown, using *N. benthamiana rdr6* CRISPR/Cas mutant plants, that RDR6 is indispensable for the amplification into secondary sRNAs of the primary ck-RNAi signals that were translocated from a root-residing beneficial fungal strain to the host plant, eventually allowing the onset of systemic silencing (Kellari *et al.*, 2025). It appears that the primary ck-RNAi signal does not suffice (e.g. due to degradation or simply dilution) to mediate systemic RNAi in the host (Fig. 1B, D). However, upon RDR6-based amplification, the secondary sRNAs undergoing a self-reinforcing loop will eventually suffice to move not only cell-to-cell through plasmodesmata but also systemically through the phloem (Fig. 1C, E). Whether RDR6-based amplification will occur depends on the nature of both the sRNA and the target mRNA. Indeed, RDR6 is ideally (but not exclusively) recruited to interactions where sRNAs having an asymmetric bulge and/or a size of 22 nt occupy the 5′ end of a transcript originating from an intronless gene (Chen *et al.*, 2010; Christie *et al.*, 2011; Manavella *et al.*, 2012; Dadami *et al.*, 2014; Uslu *et al.*, 2022). Thus, ck-RNAi does not always become systemic, but it may well do if the above criteria are met. The role of RDRs for RNAi amplification is not limited to plant hosts; fungi also encode RDRs and they may also have a role in the amplification of silencing signals, eventually increasing the efficiency of ck-RNAi (SIGS and HIGS, included). Yet, it seems that despite their presence, fungal RDRs do not display the same RNAi amplification role as plant RDRs (Song *et al.*, 2018).

### Do translocated sRNAs trigger epigenetic modifications?

At least in plants, RNAi manifests itself not only as mRNA degradation but as DNA methylation as well, with the two pathways being tightly linked (Jones *et al.*, 1999; Taochy *et al.*, 2019; Trasser *et al.*, 2024). Whether ck-RNAi could have a potential role in inducing epigenetic modifications in recipient organisms, especially in plants where the RdDM pathway is well established, remains another as yet uncharted aspect of the mechanism. The 23 nt miRNA-like Fol-milR1 released by *F. oxysporum* f.sp. *lycopersici* preferentially binds *S. lycopersicum* AGO4a to down-regulate expression of a host calcium-binding protein kinase involved in defense signaling; although cleavage of the target transcript was observed, it was unclear whether Fol-milR1 could also trigger RdDM through its association with SlAGO4a (Ji *et al.*, 2021). Loading endosymbiont sRNAs into host AGO4 proteins opens up the prospect of these driving RdDM and even transcriptional gene silencing (TGS), hence allowing long-term regulation of gene expression in the host. Such regulation would have considerable benefits in a mutualism, potentially adapting one or both partners to increase the stability of the interaction (Qiao *et al.*, 2023). Indeed, DNA methylation is essential in Arabidopsis to establish a beneficial relationship with the root-colonizing *Trichoderma atroviride* (Rebolledo-Prudencio *et al.*, 2022). The importance of DNA methylation in symbiosis is further highlighted in ectomycorrhizal symbiosis, with hypomethylation in the *Populus* sp. host associated with decreased association with the *Laccaria bicolor* mycorrhizal fungus (Vigneaud *et al.*, 2023). Solid evidence for epigenetic modifications induced upon ck-RNAi emerged from our previous work, where it was shown that sRNA translocation from a beneficial fungus triggered DNA methylation (in the CG, CHG, and CHH context) of a host reporter gene in an RDR6-dependent manner (Dalakouras *et al.*, 2023; Kellari *et al.*, 2025). Based on the above, it is reasonable to assume that the onset of epigenetic modifications such as DNA methylation and histone modifications during ck-RNAi may be more common than once thought. Should DNA methylation establishment and maintenance in ck-RNAi be confirmed, it could imply that ck-RNAi may have a pivotal role in crop epigenetic plasticity in response to environmental cues; intriguingly, such plasticity could potentially even be used to reveal cryptic diversity for breeding purposes (Dalakouras and Vlachostergios, 2021).

## Conclusions: perspectives on co-evolution

From an evolutionary standpoint, RNAi serves as a defense strategy against viruses and transposons, and its cross-kingdom extension similarly functions as defense against invading pathogens or, conversely, as a means for the pathogens to hijack the immune system of the host. It is reasonable to assume that ck-RNAi reflects a co-evolutionary arms race between plants and microbes. sRNA recipients (plants or pathogens) adapt to newly evolved sRNAs from donors (pathogens or plants, respectively) via target gene or sRNA/miRNA locus mutation, and provided that such adaptation is to the general benefit of the organism. Possible mechanisms of sRNA or miRNA evolution may range from single nucleotide polymorphisms of pre-existing loci to *de novo* emergence of new ones through inverted gene duplication events. Moreover, horizontal gene transfer (HGT), once thought to occur exclusively in prokaryotes, is now accepted also to occur in eukaryotes such as fungi and plants (Fitzpatrick, 2012; Gonçalves *et al.*, 2024; Y. Wang *et al.*, 2025). HGT has been proposed as a driving force of sRNA evolution in bacteria (Dutcher and Raghavan, 2018); whether HGT contributes to sRNA evolution in eukaryotes remains a possibility (Yang *et al.*, 2019). Interestingly during ck-RNAi, besides novel sRNA/miRNA non-coding loci, coding genes may emerge as well; indeed, microbes may develop RNAi suppressors deactivating or affecting the accumulation of plant sRNAs (Hou *et al.*, 2019; Zhu *et al.*, 2022), reminiscent of viral suppressors of RNAi which were themselves developed as a response to the RNAi antiviral strategy of the plant (Burgyán and Havelda, 2011).

sRNA exchange extends beyond antagonistic interactions to further roles during interspecies communication, since symbiotic and beneficial microorganisms also use ck-RNAi when colonizing their host plants. In a broader view, ck-RNAi seems to represent a piece of a greater co-evolution puzzle between plants and microbes (and, possibly, other organisms as well), where not only RNAs but other molecules are exchanged. As plants evolve, they recruit microbes to assist in the adaptation to available growing environments. Microbes promote plant growth and resilience, and plants, in turn, provide microbes with nutrition (e.g. root exudates) and a desirable habitat (e.g. the rhizosphere or within plant tissues), eventually resulting in the diverse and metabolically rich microbial community that exists in the rhizosphere of terrestrial plants (Lyu *et al.*, 2021). Besides sRNAs, other molecules such as peptides or metabolites participate in the molecular exchange that underlies this evolutionary crosstalk (Frantzeskakis *et al.*, 2020). Yet, as sRNAs are less complex molecular moieties, they may evolve faster in situations demanding an immediate response; for example, even the change of a nucleotide could dramatically change their biological function when needed. Whether sRNAs are indeed the molecular ‘lingua franca’, which affects organismal crosstalk, adaptation, and co-evolution remain to be demonstrated by future studies.

## References

[eraf543-B1] Adeyinka OS, Nasir IA, Tabassum B. 2023. Host-induced silencing of the *CpCHI* gene resulted in developmental abnormalities and mortality in maize stem borer (*Chilo partellus*). PLoS One 18, e0280963. 36745624 10.1371/journal.pone.0280963 PMC9901779

[eraf543-B2] Agrawal N, Dasaradhi PVN, Mohmmed A, Malhotra P, Bhatnagar RK, Mukherjee SK. 2003. RNA interference: biology, mechanism, and applications. Microbiology and Molecular Biology Reviews 67, 657–685. 14665679 10.1128/MMBR.67.4.657-685.2003 PMC309050

[eraf543-B3] Alves CS, Nogueira FTS. 2021. Plant small RNA world growing bigger: tRNA-derived fragments, longstanding players in regulatory processes. Frontiers in Molecular Biosciences 8, 638911. 34164429 10.3389/fmolb.2021.638911 PMC8215267

[eraf543-B4] Baeg K, Iwakawa H, Tomari Y. 2017. The poly(A) tail blocks RDR6 from converting self mRNAs into substrates for gene silencing. Nature Plants 3, 17036. 28319057 10.1038/nplants.2017.36

[eraf543-B5] Baldrich P, Rutter BD, Karimi HZ, Podicheti R, Meyers BC, Innes RW. 2019. Plant extracellular vesicles contain diverse small RNA species and are enriched in 10- to 17-nucleotide ‘tiny’ RNAs. The Plant Cell 31, 315–324. 30705133 10.1105/tpc.18.00872 PMC6447009

[eraf543-B6] Bartel B . 2005. MicroRNAs directing siRNA biogenesis. Nature Structural & Molecular Biology 12, 569–571. 10.1038/nsmb0705-569 15999111

[eraf543-B7] Baulcombe D . 2004. RNA silencing in plants. Nature 431, 356–363. 15372043 10.1038/nature02874

[eraf543-B8] Baulcombe DC . 2015. VIGS, HIGS and FIGS: small RNA silencing in the interactions of viruses or filamentous organisms with their plant hosts. Current Opinion in Plant Biology 26, 141–146. 26247121 10.1016/j.pbi.2015.06.007

[eraf543-B9] Betti F, Ladera-Carmona MJ, Weits DA, et al 2021. Exogenous miRNAs induce post-transcriptional gene silencing in plants. Nature Plants 7, 1379–1388. 34650259 10.1038/s41477-021-01005-w PMC8516643

[eraf543-B10] Borges F, Martienssen RA. 2015. The expanding world of small RNAs in plants. Nature Reviews. Molecular Cell Biology 16, 727–741. 26530390 10.1038/nrm4085 PMC4948178

[eraf543-B11] Borniego ML, Singla-Rastogi M, Baldrich P, Sampangi-Ramaiah MH, Zand Karimi H, McGregor M, Meyers BC, Innes RW. 2025. Diverse plant RNAs coat *Arabidopsis* leaves and are distinct from apoplastic RNAs. Proceedings of the National Academy of Sciences, USA 122, e2409090121. 10.1073/pnas.2409090121 PMC11725841 39752527

[eraf543-B12] Brodersen P, Sakvarelidze-Achard L, Bruun-Rasmussen M, Dunoyer P, Yamamoto YY, Sieburth L, Voinnet O. 2008. Widespread translational inhibition by plant miRNAs and siRNAs. Science 320, 1185–1190. 18483398 10.1126/science.1159151

[eraf543-B13] Bucher G, Scholten J, Klingler M. 2002. Parental RNAi in *Tribolium* (Coleoptera). Current Biology 12, R85–R86. 11839285 10.1016/s0960-9822(02)00666-8

[eraf543-B14] Buck AH, Coakley G, Simbari F, et al 2014. Exosomes secreted by nematode parasites transfer small RNAs to mammalian cells and modulate innate immunity. Nature Communications 5, 5488. 10.1038/ncomms6488 PMC4263141 25421927

[eraf543-B15] Burgyán J, Havelda Z. 2011. Viral suppressors of RNA silencing. Trends in Plant Science 16, 265–272. 21439890 10.1016/j.tplants.2011.02.010

[eraf543-B16] Cai Q, Qiao L, Wang M, He B, Lin F-M, Palmquist J, Huang S-D, Jin H. 2018. Plants send small RNAs in extracellular vesicles to fungal pathogen to silence virulence genes. Science 360, 1126–1129. 29773668 10.1126/science.aar4142 PMC6442475

[eraf543-B17] Carbonell A, Martínez De Alba Á-E, Flores R, Gago S. 2008. Double-stranded RNA interferes in a sequence-specific manner with the infection of representative members of the two viroid families. Virology 371, 44–53. 18028975 10.1016/j.virol.2007.09.031

[eraf543-B18] Carthew RW, Sontheimer EJ. 2009. Origins and mechanisms of miRNAs and siRNAs. Cell 136, 642–655. 19239886 10.1016/j.cell.2009.01.035 PMC2675692

[eraf543-B19] Chen A, Halilovic L, Shay J-H, Koch A, Mitter N, Jin H. 2023. Improving RNA-based crop protection through nanotechnology and insights from cross-kingdom RNA trafficking. Current Opinion in Plant Biology 76, 102441. 37696727 10.1016/j.pbi.2023.102441 PMC10777890

[eraf543-B20] Chen H-M, Chen L-T, Patel K, Li Y-H, Baulcombe DC, Wu S-H. 2010. 22-nucleotide RNAs trigger secondary siRNA biogenesis in plants. Proceedings of the National Academy of Sciences, USA 107, 15269–15274. 10.1073/pnas.1001738107 PMC2930544 20643946

[eraf543-B21] Chen T, Tian W, Shuai Q, Wen H-G, Guo H-S, Zhao J-H. 2025. Microbe-induced gene silencing of fungal gene confers efficient resistance against *Fusarium graminearum* in maize. aBIOTECH 6, 466–471. 40994436 10.1007/s42994-025-00212-9 PMC12454818

[eraf543-B22] Cheng A-P, Huang L, Oberkofler L, Johnson NR, Glodeanu A-S, Stillman K, Weiberg A. 2025. Fungal Argonaute proteins act in bidirectional cross-kingdom RNA interference during plant infection. Proceedings of the National Academy of Sciences, USA 122, e2422756122. 10.1073/pnas.2422756122 PMC12054834 40267130

[eraf543-B23] Choudry MW, Nawaz P, Jahan N, Riaz R, Ahmed B, Raza MH, Fayyaz Z, Malik K, Afzal S. 2024. RNA based gene silencing modalities to control insect and fungal plant pests—challenges and future prospects. Physiological and Molecular Plant Pathology 130, 102241.

[eraf543-B24] Christie M, Croft LJ, Carroll BJ. 2011. Intron splicing suppresses RNA silencing in Arabidopsis. The Plant Journal 68, 159–167. 21689169 10.1111/j.1365-313X.2011.04676.x

[eraf543-B25] Cui C, Wang Y, Li Y, Sun P, Jiang J, Zhou H, Liu J, Wang S. 2022. Expression of mosquito miRNAs in entomopathogenic fungus induces pathogen-mediated host RNA interference and increases fungal efficacy. Cell Reports 41, 111527. 36288711 10.1016/j.celrep.2022.111527

[eraf543-B26] Dadami E, Dalakouras A, Zwiebel M, Krczal G, Wassenegger M. 2014. An endogene-resembling transgene is resistant to DNA methylation and systemic silencing. RNA Biology 11, 934–941. 25180820 10.4161/rna.29623 PMC4179966

[eraf543-B27] Dalakouras A, Wassenegger M. 2013. Revisiting RNA-directed DNA methylation. RNA Biology 10, 453–455. 23324611 10.4161/rna.23542 PMC3672289

[eraf543-B28] Dalakouras A, Wassenegger M, Dadami E, Ganopoulos I, Pappas ML, Papadopoulou K. 2020. Genetically modified organism-free RNA interference: exogenous application of RNA molecules in plants. Plant Physiology 182, 38–50. 31285292 10.1104/pp.19.00570 PMC6945881

[eraf543-B29] Dalakouras A, Vlachostergios D. 2021. Epigenetic approaches to crop breeding: current status and perspectives. Journal of Experimental Botany 72, 5356–5371. 34017985 10.1093/jxb/erab227

[eraf543-B30] Dalakouras A, Katsaouni A, Avramidou M, Dadami E, Tsiouri O, Vasileiadis S, Makris A, Georgopoulou ME, Papadopoulou KK. 2023. A beneficial fungal root endophyte triggers systemic RNA silencing and DNA methylation of a host reporter gene. RNA Biology 20, 20–30. 36573793 10.1080/15476286.2022.2159158 PMC9809956

[eraf543-B31] Dalakouras A, Koidou V, Papadopoulou K. 2024. DsRNA-based pesticides: considerations for efficiency and risk assessment. Chemosphere 352, 141530. 38401868 10.1016/j.chemosphere.2024.141530

[eraf543-B32] Dalmadi Á, Gyula P, Bálint J, Szittya G, Havelda Z. 2019. AGO-unbound cytosolic pool of mature miRNAs in plant cells reveals a novel regulatory step at AGO1 loading. Nucleic Acids Research 47, 9803–9817. 31392979 10.1093/nar/gkz690 PMC6765109

[eraf543-B33] De Felippes FF, Waterhouse PM. 2020. The whys and wherefores of transitivity in plants. Frontiers in Plant Science 11, 579376. 32983223 10.3389/fpls.2020.579376 PMC7488869

[eraf543-B34] Dellar ER, Hill C, Melling GE, Carter DRF, Baena-Lopez LA. 2022. Unpacking extracellular vesicles: RNA cargo loading and function. Journal of Extracellular Biology 1, e40. 38939528 10.1002/jex2.40 PMC11080855

[eraf543-B35] Derbyshire M, Mbengue M, Barascud M, Navaud O, Raffaele S. 2019. Small RNAs from the plant pathogenic fungus *Sclerotinia sclerotiorum* highlight host candidate genes associated with quantitative disease resistance. Molecular Plant Pathology 20, 1279–1297. 31361080 10.1111/mpp.12841 PMC6715603

[eraf543-B36] Devers EA, Brosnan CA, Sarazin A, Albertini D, Amsler AC, Brioudes F, Jullien PE, Lim P, Schott G, Voinnet O. 2020. Movement and differential consumption of short interfering RNA duplexes underlie mobile RNA interference. Nature Plants 6, 789–799. 32632272 10.1038/s41477-020-0687-2

[eraf543-B37] Dubrovina AS, Suprun AR, Kiselev KV. 2025. Regulation of plant genes with exogenous RNAs. International Journal of Molecular Sciences 26, 6773. 40725020 10.3390/ijms26146773 PMC12295431

[eraf543-B38] Dunker F, Trutzenberg A, Rothenpieler JS, et al 2020. Oomycete small RNAs bind to the plant RNA-induced silencing complex for virulence. eLife 9, e56096. 32441255 10.7554/eLife.56096 PMC7297541

[eraf543-B39] Dutcher HA, Raghavan R. 2018. Origin, evolution, and loss of bacterial small RNAs. Microbiology Spectrum 6, 6.2.12. 10.1128/microbiolspec.rwr-0004-2017 PMC5890949 29623872

[eraf543-B40] Dutta S, Jha SK, Prabhu KV, Kumar M, Mukhopadhyay K. 2019. Leaf rust (*Puccinia triticina*) mediated RNAi in wheat (*Triticum aestivum* L.) prompting host susceptibility. Functional & Integrative Genomics 19, 437–452. 30671704 10.1007/s10142-019-00655-6

[eraf543-B41] Eldahshoury MK, Katsarou K, Farley JT, Kalantidis K, De Marcos Lousa C. 2024. Isolation of small extracellular vesicles (sEVs) from the apoplastic wash fluid of *Nicotiana benthamiana* leaves. Current Protocols 4, e70026. 39499037 10.1002/cpz1.70026 PMC11602942

[eraf543-B42] Erdmann RM, Picard CL. 2020. RNA-directed DNA methylation. PLoS Genetics 16, e1009034. 33031395 10.1371/journal.pgen.1009034 PMC7544125

[eraf543-B43] Fabian MR, Sonenberg N, Filipowicz W. 2010. Regulation of mRNA translation and stability by microRNAs. Annual Review of Biochemistry 79, 351–379. 10.1146/annurev-biochem-060308-103103 20533884

[eraf543-B44] Fan J, Quan W, Li G-B, et al 2020. circRNAs are involved in the rice–*Magnaporthe oryzae* interaction. Plant Physiology 182, 272–286. 31628150 10.1104/pp.19.00716 PMC6945833

[eraf543-B45] Fan L, Zhang C, Gao B, Zhang Y, Stewart E, Jez J, Nakajima K, Chen X. 2022. Microtubules promote the non-cell autonomous action of microRNAs by inhibiting their cytoplasmic loading onto ARGONAUTE1 in Arabidopsis. Developmental Cell 57, 995–1008. 35429434 10.1016/j.devcel.2022.03.015 PMC9056376

[eraf543-B46] Fang X, Qi Y. 2016. RNAi in plants: an Argonaute-centered view. The Plant Cell 28, 272–285. 26869699 10.1105/tpc.15.00920 PMC4790879

[eraf543-B47] Fei Y, Nyikó T, Molnar A. 2021. Non-perfectly matching small RNAs can induce stable and heritable epigenetic modifications and can be used as molecular markers to trace the origin and fate of silencing RNAs. Nucleic Acids Research 49, 1900–1913. 33524108 10.1093/nar/gkab023 PMC7913690

[eraf543-B48] Fire A, Albertson D, Harrison SW, Moerman DG. 1991. Production of antisense RNA leads to effective and specific inhibition of gene expression in *C. elegans* muscle. Development 113, 503–514. 1782862 10.1242/dev.113.2.503

[eraf543-B49] Fitzpatrick DA . 2012. Horizontal gene transfer in fungi. FEMS Microbiology Letters 329, 1–8. 22112233 10.1111/j.1574-6968.2011.02465.x

[eraf543-B50] Franco-Zorrilla JM, Valli A, Todesco M, Mateos I, Puga MI, Rubio-Somoza I, Leyva A, Weigel D, García JA, Paz-Ares J. 2007. Target mimicry provides a new mechanism for regulation of microRNA activity. Nature Genetics 39, 1033–1037. 17643101 10.1038/ng2079

[eraf543-B51] Frantzeskakis L, Di Pietro A, Rep M, Schirawski J, Wu C, Panstruga R. 2020. Rapid evolution in plant–microbe interactions—a molecular genomics perspective. New Phytologist 225, 1134–1142. 31134629 10.1111/nph.15966

[eraf543-B52] Gallego-Bartolomé J . 2020. DNA methylation in plants: mechanisms and tools for targeted manipulation. New Phytologist 227, 38–44. 32159848 10.1111/nph.16529

[eraf543-B53] Gauthier MA, Shand K, Hayashi S, Waterhouse PM, Barrero RA, De Felippes FF. 2025. MicroRNA-induced gene silencing (MIGS): a tool for multi-gene silencing and targeting viruses in plants. Plant Biotechnology Journal doi: 10.1111/pbi.70401. PMC12946496 41053986

[eraf543-B54] Gazzani S, Lawrenson T, Woodward C, Headon D, Sablowski R. 2004. A link between mRNA turnover and RNA interference in *Arabidopsis*. Science 306, 1046–1048. 15528448 10.1126/science.1101092

[eraf543-B55] Gonçalves C, Hittinger CT, Rokas A. 2024. Horizontal gene transfer in fungi and its ecological importance. In: Hsueh Y-P, Blackwell M, eds. The mycota. Fungal associations. Cham: Springer International Publishing, 59–81.

[eraf543-B56] Gonzalo L, Gagliardi D, Zlauvinen C, et al 2025. The nuclear pore complex acts as a hub for pri-miRNA transcription and processing in plants. Nucleic Acids Research 53, gkaf885. 40966524 10.1093/nar/gkaf885 PMC12448844

[eraf543-B57] Govindarajulu M, Epstein L, Wroblewski T, Michelmore RW. 2015. Host-induced gene silencing inhibits the biotrophic pathogen causing downy mildew of lettuce. Plant Biotechnology Journal 13, 875–883. 25487781 10.1111/pbi.12307

[eraf543-B58] Gu K-X, Song X-S, Xiao X-M, Duan X-X, Wang J-X, Duan Y-B, Hou Y-P, Zhou M-G. 2019. A *β*2*-tubulin* dsRNA derived from *Fusarium asiaticum* confers plant resistance to multiple phytopathogens and reduces fungicide resistance. Pesticide Biochemistry and Physiology 153, 36–46. 30744895 10.1016/j.pestbp.2018.10.005

[eraf543-B59] Hagiwara K, Katsuda T, Gailhouste L, Kosaka N, Ochiya T. 2015. Commitment of Annexin A2 in recruitment of microRNAs into extracellular vesicles. FEBS Letters 589, 4071–4078. 26632510 10.1016/j.febslet.2015.11.036

[eraf543-B60] Halder LD, Babych S, Palme DI, et al 2022. *Candida albicans* induces cross-kingdom miRNA trafficking in human monocytes to promote fungal growth. mBio 13, e0356321. 10.1128/mbio.03563-21 PMC8822622 35132877

[eraf543-B61] He B, Cai Q, Qiao L, Huang C-Y, Wang S, Miao W, Ha T, Wang Y, Jin H. 2021. RNA-binding proteins contribute to small RNA loading in plant extracellular vesicles. Nature Plants 7, 342–352. 33633358 10.1038/s41477-021-00863-8 PMC7979528

[eraf543-B62] He B, Wang H, Liu G, Chen A, Calvo A, Cai Q, Jin H. 2023. Fungal small RNAs ride in extracellular vesicles to enter plant cells through clathrin-mediated endocytosis. Nature Communications 14, 4383. 10.1038/s41467-023-40093-4 PMC10359353 37474601

[eraf543-B63] Hendrix B, Zheng W, Bauer MJ, et al 2021. Topically delivered 22 nt siRNAs enhance RNAi silencing of endogenous genes in two species. Planta 254, 60. 34448043 10.1007/s00425-021-03708-y PMC8390415

[eraf543-B64] Hou Y, Zhai Y, Feng L, et al 2019. A *Phytophthora* effector suppresses trans-kingdom RNAi to promote disease susceptibility. Cell Host & Microbe 25, 153–165. 30595554 10.1016/j.chom.2018.11.007 PMC9208300

[eraf543-B65] Hu M, Han Y, Zhang X, Tian S, Shang Z, Yuan Z, He L. 2025. Extracellular vesicles for targeted drug delivery: advances in surface modification strategies and therapeutic applications. Journal of Translational Medicine 23, 1028. 41029680 10.1186/s12967-025-07077-y PMC12486671

[eraf543-B66] Iwakawa H, Tomari Y. 2022. Life of RISC: formation, action, and degradation of RNA-induced silencing complex. Molecular Cell 82, 30–43. 34942118 10.1016/j.molcel.2021.11.026

[eraf543-B67] Jahan SN, Åsman AKM, Corcoran P, Fogelqvist J, Vetukuri RR, Dixelius C. 2015. Plant-mediated gene silencing restricts growth of the potato late blight pathogen *Phytophthora infestans*. Journal of Experimental Botany 66, 2785–2794. 25788734 10.1093/jxb/erv094 PMC4986879

[eraf543-B68] Ji H, Mao H, Li S, Feng T, Zhang Z, Cheng L, Luo S, Borkovich KA, Ouyang S. 2021. *Fol*-milR1, a pathogenicity factor of *Fusarium oxysporum*, confers tomato wilt disease resistance by impairing host immune responses. New Phytologist 232, 705–718. 33960431 10.1111/nph.17436 PMC8518127

[eraf543-B69] Ji L, Chen X. 2012. Regulation of small RNA stability: methylation and beyond. Cell Research 22, 624–636. 22410795 10.1038/cr.2012.36 PMC3317568

[eraf543-B70] Jiao J, Peng D. 2018. Wheat microRNA1023 suppresses invasion of *Fusarium graminearum* via targeting and silencing *FGSG_03101*. Journal of Plant Interactions 13, 514–521.

[eraf543-B71] Joga MR, Zotti MJ, Smagghe G, Christiaens O. 2016. RNAi efficiency, systemic properties, and novel delivery methods for pest insect control: what we know so far. Frontiers in Physiology 7, 553. 27909411 10.3389/fphys.2016.00553 PMC5112363

[eraf543-B72] Jones L, Hamilton AJ, Voinnet O, Thomas CL, Maule AJ, Baulcombe DC. 1999. RNA–DNA interactions and DNA methylation in post-transcriptional gene silencing. The Plant Cell 11, 2291–2301. 10590159 10.1105/tpc.11.12.2291 PMC144133

[eraf543-B73] Jouravleva K, Zamore PD. 2025. A guide to the biogenesis and functions of endogenous small non-coding RNAs in animals. Nature Reviews. Molecular Cell Biology 26, 347–370. 39856370 10.1038/s41580-024-00818-9

[eraf543-B74] Kalyandurg PB, Sundararajan P, Dubey M, Ghadamgahi F, Zahid MA, Whisson SC, Vetukuri RR. 2021. Spray-induced gene silencing as a potential tool to control potato late blight disease. Phytopathology 111, 2168–2175. 33973799 10.1094/PHYTO-02-21-0054-SC

[eraf543-B75] Keam S, Hutvagner G. 2015. tRNA-derived fragments (tRFs): emerging new roles for an ancient RNA in the regulation of gene expression. Life 5, 1638–1651. 26703738 10.3390/life5041638 PMC4695841

[eraf543-B76] Kellari LM, Dalakouras A, Tsiouri O, Vletsos P, Katsaouni A, Uslu VV, Papadopoulou KK. 2025. Cross-kingdom RNAi induced by a beneficial endophytic fungus to its host requires transitivity and amplification of silencing signals. Plant Biology 27, 504–514. 40377112 10.1111/plb.70026 PMC12096064

[eraf543-B77] Kettles GJ, Hofinger BJ, Hu P, Bayon C, Rudd JJ, Balmer D, Courbot M, Hammond-Kosack KE, Scalliet G, Kanyuka K. 2019. sRNA profiling combined with gene function analysis reveals a lack of evidence for cross-kingdom RNAi in the wheat–*Zymoseptoria tritici* pathosystem. Frontiers in Plant Science 10, 892. 31333714 10.3389/fpls.2019.00892 PMC6620828

[eraf543-B78] Knip M, Constantin ME, Thordal-Christensen H. 2014. Trans-kingdom cross-talk: small RNAs on the move. PLoS Genetics 10, e1004602. 25188222 10.1371/journal.pgen.1004602 PMC4154666

[eraf543-B79] Kobayashi K, Zambryski P. 2007. RNA silencing and its cell-to-cell spread during Arabidopsis embryogenesis. The Plant Journal 50, 597–604. 17419839 10.1111/j.1365-313X.2007.03073.x

[eraf543-B80] Koch A, Kogel K-H. 2014. New wind in the sails: improving the agronomic value of crop plants through RNAi-mediated gene silencing. Plant Biotechnology Journal 12, 821–831. 25040343 10.1111/pbi.12226

[eraf543-B81] Koch A, Biedenkopf D, Furch A, et al 2016. An RNAi-based control of *Fusarium graminearum* infections through spraying of long dsRNAs involves a plant passage and is controlled by the fungal silencing machinery. PLoS Pathogens 12, e1005901. 27737019 10.1371/journal.ppat.1005901 PMC5063301

[eraf543-B82] Koch A, Höfle L, Werner BT, Imani J, Schmidt A, Jelonek L, Kogel K. 2019. SIGS vs HIGS: a study on the efficacy of two dsRNA delivery strategies to silence *Fusarium FgCYP51* genes in infected host and non-host plants. Molecular Plant Pathology 20, 1636–1644. 31603277 10.1111/mpp.12866 PMC6859480

[eraf543-B83] Koch A, Wassenegger M. 2021. Host-induced gene silencing—mechanisms and applications. New Phytologist 231, 54–59. 33774815 10.1111/nph.17364

[eraf543-B84] Koch BL, Rutter BD, Borniego ML, Singla-Rastogi M, Gardner DM, Innes RW. 2025. Arabidopsis produces distinct subpopulations of extracellular vesicles that respond differentially to biotic stress, altering growth and infectivity of a fungal pathogen. Journal of Extracellular Vesicles 14, e70090. 40415221 10.1002/jev2.70090 PMC12104214

[eraf543-B85] Koeppen K, Hampton TH, Jarek M, et al 2016. A novel mechanism of host–pathogen interaction through sRNA in bacterial outer membrane vesicles. PLOS Pathogens 12, e1005672. 27295279 10.1371/journal.ppat.1005672 PMC4905634

[eraf543-B86] Koeppen K, Nymon A, Barnaby R, et al 2021. Let-7b-5p in vesicles secreted by human airway cells reduces biofilm formation and increases antibiotic sensitivity of *P. aeruginosa*. Proceedings of the National Academy of Sciences, USA 118, e2105370118. 10.1073/pnas.2105370118 PMC8285967 34260396

[eraf543-B87] Kong L, Shi X, Chen D, et al 2022. Host-induced silencing of a nematode chitin synthase gene enhances resistance of soybeans to both pathogenic *Heterodera glycines* and *Fusarium oxysporum*. Plant Biotechnology Journal 20, 809–811. 35301818 10.1111/pbi.13808 PMC9055809

[eraf543-B88] Kusch S, Frantzeskakis L, Thieron H, Panstruga R. 2018. Small RNAs from cereal powdery mildew pathogens may target host plant genes. Fungal Biology 122, 1050–1063. 30342621 10.1016/j.funbio.2018.08.008

[eraf543-B89] Kwon S, Rupp O, Brachmann A, Blum CF, Kraege A, Goesmann A, Feldbrügge M. 2021. mRNA inventory of extracellular vesicles from *Ustilago maydis*. Journal of Fungi 7, 562. 34356940 10.3390/jof7070562 PMC8306574

[eraf543-B90] LaMonte G, Philip N, Reardon J, et al 2012. Translocation of sickle cell erythrocyte microRNAs into *Plasmodium falciparum* inhibits parasite translation and contributes to malaria resistance. Cell Host & Microbe 12, 187–199. 22901539 10.1016/j.chom.2012.06.007 PMC3442262

[eraf543-B91] Laurie JD, Linning R, Bakkeren G. 2008. Hallmarks of RNA silencing are found in the smut fungus *Ustilago hordei* but not in its close relative *Ustilago maydis*. Current Genetics 53, 49–58. 18060405 10.1007/s00294-007-0165-7

[eraf543-B92] Li D, Li Z, Wu J, Tang Z, Xie F, Chen D, Lin H, Li Y. 2022. Analysis of outer membrane vesicles indicates that glycerophospholipid metabolism contributes to early symbiosis between *Sinorhizobium fredii* HH103 and soybean. Molecular Plant-Microbe Interactions 35, 311–322. 34978930 10.1094/MPMI-11-21-0288-R

[eraf543-B93] Liang J, Wang J, Wang K, Feng H, Huang L. 2024. VmRDR2 of *Valsa mali* mediates the generation of *VmR2*-siR1 that suppresses apple resistance by RNA interference. New Phytologist 243, 1154–1171. 38822646 10.1111/nph.19867

[eraf543-B94] Lyu D, Msimbira LA, Nazari M, et al 2021. The coevolution of plants and microbes underpins sustainable agriculture. Microorganisms 9, 1036. 34065848 10.3390/microorganisms9051036 PMC8151373

[eraf543-B95] Maas SLN, Breakefield XO, Weaver AM. 2017. Extracellular vesicles: unique intercellular delivery vehicles. Trends in Cell Biology 27, 172–188. 27979573 10.1016/j.tcb.2016.11.003 PMC5318253

[eraf543-B96] Mahanty B, Mishra R, Joshi RK. 2023. Cross-kingdom small RNA communication between plants and fungal phytopathogens—recent updates and prospects for future agriculture. RNA Biology 20, 109–119. 36988190 10.1080/15476286.2023.2195731 PMC10062216

[eraf543-B97] Mahto BK, Singh A, Pareek M, Rajam MV, Dhar-Ray S, Reddy PM. 2020. Host-induced silencing of the *Colletotrichum gloeosporioides conidial morphology 1* gene (*CgCOM1*) confers resistance against anthracnose disease in chilli and tomato. Plant Molecular Biology 104, 381–395. 32803478 10.1007/s11103-020-01046-3

[eraf543-B98] Mallory AC, Elmayan T, Vaucheret H. 2008. MicroRNA maturation and action—the expanding roles of ARGONAUTEs. Current Opinion in Plant Biology 11, 560–566. 18691933 10.1016/j.pbi.2008.06.008

[eraf543-B99] Manavella PA, Koenig D, Weigel D. 2012. Plant secondary siRNA production determined by microRNA-duplex structure. Proceedings of the National Academy of Sciences, USA 109, 2461–2466. 10.1073/pnas.1200169109 PMC3289316 22308502

[eraf543-B100] Mani V, Reddy CS, Lee S-K, Park S, Ko H-R, Kim D-G, Hahn B-S. 2020. Chitin biosynthesis inhibition of *Meloidogyne incognita* by RNAi-mediated gene silencing increases resistance to transgenic tobacco plants. International Journal of Molecular Sciences 21, 6626. 32927773 10.3390/ijms21186626 PMC7555284

[eraf543-B101] Martínez De Alba AE, Elvira-Matelot E, Vaucheret H. 2013. Gene silencing in plants: a diversity of pathways. Biochimica et Biophysica Acta 1829, 1300–1308. 24185199 10.1016/j.bbagrm.2013.10.005

[eraf543-B102] Matzke MA, Mosher RA. 2014. RNA-directed DNA methylation: an epigenetic pathway of increasing complexity. Nature Reviews. Genetics 15, 394–408. 10.1038/nrg3683 24805120

[eraf543-B103] Melnyk CW, Molnar A, Baulcombe DC. 2011. Intercellular and systemic movement of RNA silencing signals: intercellular and systemic movement of RNA silencing signals. The EMBO Journal 30, 3553–3563. 21878996 10.1038/emboj.2011.274 PMC3181474

[eraf543-B104] Mewalal R, Yin H, Hu R, Jawdy S, Vion P, Tuskan GA, Le Tacon F, Labbé JL, Yang X. 2019. Identification of *Populus* small RNAs responsive to mutualistic interactions with mycorrhizal fungi, *Laccaria bicolor* and *Rhizophagus irregularis*. Frontiers in Microbiology 10, 515. 30936859 10.3389/fmicb.2019.00515 PMC6431645

[eraf543-B105] Mi S, Cai T, Hu Y, et al 2008. Sorting of small RNAs into Arabidopsis Argonaute complexes is directed by the 5′ terminal nucleotide. Cell 133, 116–127. 18342361 10.1016/j.cell.2008.02.034 PMC2981139

[eraf543-B106] Mittelbrunn M, Sánchez-Madrid F. 2012. Intercellular communication: diverse structures for exchange of genetic information. Nature Reviews. Molecular Cell Biology 13, 328–335. 22510790 10.1038/nrm3335 PMC3738855

[eraf543-B107] Mitter N, Worrall EA, Robinson KE, Li P, Jain RG, Taochy C, Fletcher SJ, Carroll BJ, Lu GQ, Xu ZP. 2017. Clay nanosheets for topical delivery of RNAi for sustained protection against plant viruses. Nature Plants 3, 16207. 28067898 10.1038/nplants.2016.207

[eraf543-B108] Mueth NA, Hulbert SH. 2022. Small RNAs target native and cross-kingdom transcripts on both sides of the wheat stripe rust interaction. Genomics 114, 110526. 36427746 10.1016/j.ygeno.2022.110526

[eraf543-B109] Murtaza S, Tabassum B, Tariq M, Riaz S, Yousaf I, Jabbar B, Khan A, Samuel AO, Zameer M, Nasir IA. 2022. Silencing a *Myzus persicae* macrophage inhibitory factor by plant-mediated RNAi induces enhanced aphid mortality coupled with boosted RNAi efficacy in transgenic potato lines. Molecular Biotechnology 64, 1152–1163. 35460447 10.1007/s12033-022-00498-w

[eraf543-B110] Napoli C, Lemieux C, Jorgensen R. 1990. Introduction of a chimeric chalcone synthase gene into Petunia results in reversible co-suppression of homologous genes in trans. The Plant Cell 2, 279–289. 12354959 10.1105/tpc.2.4.279 PMC159885

[eraf543-B111] Niehl A, Soininen M, Poranen MM, Heinlein M. 2018. Synthetic biology approach for plant protection using dsRNA. Plant Biotechnology Journal 16, 1679–1687. 29479789 10.1111/pbi.12904 PMC6097125

[eraf543-B112] Nien Y-C, Vanek A, Axtell MJ. 2024. *Trans*-species mobility of RNA interference between plants and associated organisms. Plant & Cell Physiology 65, 694–703. 38288670 10.1093/pcp/pcae012

[eraf543-B113] Niu D, Hamby R, Sanchez JN, Cai Q, Yan Q, Jin H. 2021. RNAs—a new frontier in crop protection. Current Opinion in Biotechnology 70, 204–212. 34217122 10.1016/j.copbio.2021.06.005 PMC8957476

[eraf543-B114] Nowara D, Gay A, Lacomme C, Shaw J, Ridout C, Douchkov D, Hensel G, Kumlehn J, Schweizer P. 2010. HIGS: host-induced gene silencing in the obligate biotrophic fungal pathogen *Blumeria graminis*. The Plant Cell 22, 3130–3141. 20884801 10.1105/tpc.110.077040 PMC2965548

[eraf543-B115] Omolehin O, Raruang Y, Hu D, et al 2024. Host-induced gene silencing of the *Aspergillus flavus O*-methyl transferase gene enhanced maize aflatoxin resistance. Toxins 17, 8. 39852961 10.3390/toxins17010008 PMC11769010

[eraf543-B116] Pérez CEB, Cabral GB, Aragão FJL. 2021. Host-induced gene silencing for engineering resistance to *Fusarium* in soybean. Plant Pathology 70, 417–425.

[eraf543-B117] Perez MG, Gillan V, Anderson WM, Gerbe F, Herbert F, McNeilly TN, Maizels RM, Jay P, Devaney E, Britton C. 2025. A secreted helminth microRNA suppresses gastrointestinal cell differentiation required for innate immunity. Frontiers in Immunology 16, 1558132. 40213548 10.3389/fimmu.2025.1558132 PMC11983496

[eraf543-B118] Piombo E, Vetukuri RR, Tzelepis G, Funck Jensen D, Karlsson M, Dubey M. 2024. Small RNAs: a new paradigm in fungal–fungal interactions used for biocontrol. Fungal Biology Reviews 48, 100356.

[eraf543-B119] Porquier A, Tisserant C, Salinas F, Glassl C, Wange L, Enard W, Hauser A, Hahn M, Weiberg A. 2021. Retrotransposons as pathogenicity factors of the plant pathogenic fungus *Botrytis cinerea*. Genome Biology 22, 225. 34399815 10.1186/s13059-021-02446-4 PMC8365987

[eraf543-B120] Qiao L, Lan C, Capriotti L, et al 2021. Spray-induced gene silencing for disease control is dependent on the efficiency of pathogen RNA uptake. Plant Biotechnology Journal 19, 1756–1768. 33774895 10.1111/pbi.13589 PMC8428832

[eraf543-B121] Qiao SA, Gao Z, Roth R. 2023. A perspective on cross-kingdom RNA interference in mutualistic symbioses. New Phytologist 240, 68–79. 37452489 10.1111/nph.19122 PMC10952549

[eraf543-B122] Ravet A, Zervudacki J, Singla-Rastogi M, et al 2025. Vesicular and non-vesicular extracellular small RNAs direct gene silencing in a plant-interacting bacterium. Nature Communications 16, 3533. 10.1038/s41467-025-57908-1 PMC11997071 40229238

[eraf543-B123] Rebolledo-Prudencio OG, Estrada-Rivera M, Dautt-Castro M, Arteaga-Vazquez MA, Arenas-Huertero C, Rosendo-Vargas MM, Jin H, Casas-Flores S. 2022. The small RNA-mediated gene silencing machinery is required in Arabidopsis for stimulation of growth, systemic disease resistance, and suppression of the nitrile-specifier gene NSP4 by *Trichoderma atroviride*. The Plant Journal 109, 873–890. 34807478 10.1111/tpj.15599

[eraf543-B124] Ren B, Wang X, Duan J, Ma J. 2019. Rhizobial tRNA-derived small RNAs are signal molecules regulating plant nodulation. Science 365, 919–922. 31346137 10.1126/science.aav8907

[eraf543-B125] Rodrigues TB, Mishra SK, Sridharan K, et al 2021. First sprayable double-stranded RNA-based biopesticide product targets proteasome subunit beta type-5 in Colorado potato beetle (*Leptinotarsa decemlineata*). Frontiers in Plant Science 12, 728652. 34887882 10.3389/fpls.2021.728652 PMC8650841

[eraf543-B126] Romano N, Macino G. 1992. Quelling: transient inactivation of gene expression in *Neurospora crassa* by transformation with homologous sequences. Molecular Microbiology 6, 3343–3353. 1484489 10.1111/j.1365-2958.1992.tb02202.x

[eraf543-B127] Sall IM, Flaviu TA. 2023. Plant and mammalian-derived extracellular vesicles: a new therapeutic approach for the future. Frontiers in Bioengineering and Biotechnology 11, 1215650. 37781539 10.3389/fbioe.2023.1215650 PMC10534050

[eraf543-B128] Schwartz SH, Hendrix B, Hoffer P, Sanders RA, Zheng W. 2020. Carbon dots for efficient small interfering RNA delivery and gene silencing in plants. Plant Physiology 184, 647–657. 32764133 10.1104/pp.20.00733 PMC7536711

[eraf543-B129] Šečić E, Zanini S, Wibberg D, et al 2021. A novel plant–fungal association reveals fundamental sRNA and gene expression reprogramming at the onset of symbiosis. BMC Biology 19, 171. 34429124 10.1186/s12915-021-01104-2 PMC8385953

[eraf543-B130] Shahid S, Kim G, Johnson NR, et al 2018. MicroRNAs from the parasitic plant *Cuscuta campestris* target host messenger RNAs. Nature 553, 82–85. 29300014 10.1038/nature25027

[eraf543-B131] Silvestri A, Fiorilli V, Miozzi L, Accotto GP, Turina M, Lanfranco L. 2019. In silico analysis of fungal small RNA accumulation reveals putative plant mRNA targets in the symbiosis between an arbuscular mycorrhizal fungus and its host plant. BMC Genomics 20, 169. 30832582 10.1186/s12864-019-5561-0 PMC6399891

[eraf543-B132] Silvestri A, Ledford WC, Fiorilli V, et al 2025. A fungal sRNA silences a host plant transcription factor to promote arbuscular mycorrhizal symbiosis. New Phytologist 246, 924–935. 39555692 10.1111/nph.20273 PMC11982788

[eraf543-B133] Song X, Gu K, Duan X, Xiao X, Hou Y, Duan Y, Wang J, Yu N, Zhou M. 2018. Secondary amplification of siRNA machinery limits the application of spray-induced gene silencing. Molecular Plant Pathology 19, 2543–2560. 30027625 10.1111/mpp.12728 PMC6638038

[eraf543-B134] Soutschek J, Akinc A, Bramlage B, et al 2004. Therapeutic silencing of an endogenous gene by systemic administration of modified siRNAs. Nature 432, 173–178. 15538359 10.1038/nature03121

[eraf543-B135] Tang Y, Wu S, He H, Gao Q, Ding W, Xue J, Qiu L, Li Y. 2024. The CsmiR1579–CsKr-h1 module mediates rice stem borer development and reproduction: an effective target for transgenic insect-resistant rice. International Journal of Biological Macromolecules 254, 127752. 38287594 10.1016/j.ijbiomac.2023.127752

[eraf543-B136] Taochy C, Yu A, Bouché N, Bouteiller N, Elmayan T, Dressel U, Carroll BJ, Vaucheret H. 2019. Post-transcriptional gene silencing triggers dispensable DNA methylation in gene body in Arabidopsis. Nucleic Acids Research 47, 9104–9114. 31372641 10.1093/nar/gkz636 PMC6753489

[eraf543-B137] Tardin-Coelho R, Fletcher S, Manzie N, Gunasekara SN, Fidelman P, Mitter N, Ashworth P. 2025. A systematic review on public perceptions of RNAi-based biopesticides: developing social licence to operate. Npj Sustainable Agriculture 3, 15.

[eraf543-B138] Thompson MC, Feng H, Wuchty S, Wilson ACC. 2019. The green peach aphid gut contains host plant microRNAs identified by comprehensive annotation of *Brassica oleracea* small RNA data. Scientific Reports 9, 18904. 31827121 10.1038/s41598-019-54488-1 PMC6906386

[eraf543-B139] Tomoyasu Y, Miller SC, Tomita S, Schoppmeier M, Grossmann D, Bucher G. 2008. Exploring systemic RNA interference in insects: a genome-wide survey for RNAi genes in *Tribolium*. Genome Biology 9, R10. 18201385 10.1186/gb-2008-9-1-r10 PMC2395250

[eraf543-B140] Toubarro D, Kenney E, Heryanto C, Mallick S, Simões N, Eleftherianos I. 2025. *Heterorhabditis bacteriophora* extracellular vesicles alter the innate immune signaling in *Drosophila melanogaster*. Genes 16, 613. 40565505 10.3390/genes16060613 PMC12191515

[eraf543-B141] Tournier B, Tabler M, Kalantidis K. 2006. Phloem flow strongly influences the systemic spread of silencing in GFP *Nicotiana benthamiana* plants. The Plant Journal 47, 383–394. 16771840 10.1111/j.1365-313X.2006.02796.x

[eraf543-B142] Trasser M, Bohl-Viallefond G, Barragán-Borrero V, Diezma-Navas L, Loncsek L, Nordborg M, Marí-Ordóñez A. 2024. PTGS is dispensable for the initiation of epigenetic silencing of an active transposon in Arabidopsis. EMBO Reports 25, 5780–5809. 39511423 10.1038/s44319-024-00304-5 PMC11624286

[eraf543-B143] Uslu VV, Dalakouras A, Steffens VA, Krczal G, Wassenegger M. 2022. High-pressure sprayed siRNAs influence the efficiency but not the profile of transitive silencing. The Plant Journa 109, 1199–1212. 10.1111/tpj.15625 34882879

[eraf543-B144] Valadi H, Ekström K, Bossios A, Sjöstrand M, Lee JJ, Lötvall JO. 2007. Exosome-mediated transfer of mRNAs and microRNAs is a novel mechanism of genetic exchange between cells. Nature Cell Biology 9, 654–659. 17486113 10.1038/ncb1596

[eraf543-B145] Van Kleeff PJM, Galland M, Schuurink RC, Bleeker PM. 2016. Small RNAs from *Bemisia tabaci* are transferred to *Solanum lycopersicum* phloem during feeding. Frontiers in Plant Science 7, 1759. 27933079 10.3389/fpls.2016.01759 PMC5121246

[eraf543-B146] Vatanparast M, Merkel L, Amari K. 2024. Exogenous application of dsRNA in plant protection: efficiency, safety concerns and risk assessment. International Journal of Molecular Sciences 25, 6530. 38928236 10.3390/ijms25126530 PMC11204322

[eraf543-B147] Vaucheret H, Voinnet O. 2024. The plant siRNA landscape. The Plant Cell 36, 246–275. 37772967 10.1093/plcell/koad253 PMC10827316

[eraf543-B148] Venkatesh J, Kim SJ, Siddique MI, Kim JH, Lee SH, Kang B-C. 2023. CopE and TLR6 RNAi-mediated tomato resistance to western flower thrips. Journal of Integrative Agriculture 22, 471–480.

[eraf543-B149] Vigneaud J, Kohler A, Sow MD, et al 2023. DNA hypomethylation of the host tree impairs interaction with mutualistic ectomycorrhizal fungus. New Phytologist 238, 2561–2577. 36807327 10.1111/nph.18734

[eraf543-B150] Voinnet O . 2009. Origin, biogenesis, and activity of plant microRNAs. Cell 136, 669–687. 19239888 10.1016/j.cell.2009.01.046

[eraf543-B151] Voinnet O . 2022. Revisiting small RNA movement in plants. Nature Reviews, Molecular Cell Biology 23, 163–164. 10.1038/s41580-022-00455-0 35087242

[eraf543-B152] Voinnet O . 2025. Three decades of mobile RNA silencing within plants: what have we learnt? Journal of Experimental Botany 77, 799–817. 10.1093/jxb/eraf312 PMC12835823 40629501

[eraf543-B153] Wang B, Sun Y, Song N, Zhao M, Liu R, Feng H, Wang X, Kang Z. 2017. *Puccinia striiformis* f. sp. *tritici* microRNA-like RNA 1 (*Pst*-milR1), an important pathogenicity factor of *Pst*, impairs wheat resistance to *Pst* by suppressing the wheat pathogenesis-related 2 gene. New Phytologist 215, 338–350. 28464281 10.1111/nph.14577

[eraf543-B154] Wang M, Weiberg A, Dellota E, Yamane D, Jin H. 2017. Botrytis small RNA *Bc*-siR37 suppresses plant defense genes by cross-kingdom RNAi. RNA Biology 14, 421–428. 28267415 10.1080/15476286.2017.1291112 PMC5411126

[eraf543-B155] Wang M, Dean RA. 2020. Movement of small RNAs in and between plants and fungi. Molecular Plant Pathology 21, 589–601. 32027079 10.1111/mpp.12911 PMC7060135

[eraf543-B156] Wang M, Wu L, Mei Y, Zhao Y, Ma Z, Zhang X, Chen Y. 2020. Host-induced gene silencing of multiple genes of *Fusarium graminearum* enhances resistance to Fusarium head blight in wheat. Plant Biotechnology Journal 18, 2373–2375. 32436275 10.1111/pbi.13401 PMC7680546

[eraf543-B157] Wang Q, Ye Y, Wang L, Guan Y, Wang S, Wang Z, Sun H, Smith SM, Huang J. 2025. Independent horizontal transfer of genes encoding α/β-hydrolases with strigolactone binding and hydrolytic activities from bacteria to fungi and plants. Molecular Plant 18, 1949–1961. 41039777 10.1016/j.molp.2025.09.021

[eraf543-B158] Wang S, He B, Wu H, Cai Q, Ramírez-Sánchez O, Abreu-Goodger C, Birch PRJ, Jin H. 2024. Plant mRNAs move into a fungal pathogen via extracellular vesicles to reduce infection. Cell Host & Microbe 32, 93–105.e6. 38103543 10.1016/j.chom.2023.11.020 PMC10872371

[eraf543-B159] Wang W, Zhang F, Cui J, Chen D, Liu Z, Hou J, Zhang R, Liu T. 2021. Identification of microRNA-like RNAs from *Trichoderma asperellum* DQ-1 during its interaction with tomato roots using bioinformatic analysis and high-throughput sequencing. PLoS One 16, e0254808. 34293017 10.1371/journal.pone.0254808 PMC8297844

[eraf543-B160] Wang Y, Cui C, Wang G, Li Y, Wang S. 2021. Insects defend against fungal infection by employing microRNAs to silence virulence-related genes. Proceedings of the National Academy of Sciences, USA 118, e2023802118. 10.1073/pnas.2023802118 PMC8126844 33941699

[eraf543-B161] Wang Y, Duan Y, Liu M, et al 2025. Target gene selection for sprayable dsRNA-based biopesticide against *Tetranychus urticae* Koch (Acari: Tetranychidae). Pest Management Science 81, 3055–3065. 39887845 10.1002/ps.8675 PMC12074632

[eraf543-B162] Wassenegger M, Krczal G. 2006. Nomenclature and functions of RNA-directed RNA polymerases. Trends in Plant Science 11, 142–151. 16473542 10.1016/j.tplants.2006.01.003

[eraf543-B163] Weiberg A, Wang M, Lin F-M, Zhao H, Zhang Z, Kaloshian I, Huang H-D, Jin H. 2013. Fungal small RNAs suppress plant immunity by hijacking host RNA interference pathways. Science 342, 118–123. 24092744 10.1126/science.1239705 PMC4096153

[eraf543-B164] Wen H-G, Zhao J-H, Zhang B-S, Gao F, Wu X-M, Yan Y-S, Zhang J, Guo H-S. 2023. Microbe-induced gene silencing boosts crop protection against soil-borne fungal pathogens. Nature Plants 9, 1409–1418. 37653339 10.1038/s41477-023-01507-9

[eraf543-B165] Winston WM, Molodowitch C, Hunter CP. 2002. Systemic RNAi in *C. elegans* requires the putative transmembrane protein SID-1. Science 295, 2456–2459. 11834782 10.1126/science.1068836

[eraf543-B166] Winston WM, Sutherlin M, Wright AJ, Feinberg EH, Hunter CP. 2007. *Caenorhabditis elegans* SID-2 is required for environmental RNA interference. Proceedings of the National Academy of Sciences, USA 104, 10565–10570. 10.1073/pnas.0611282104 PMC1965553 17563372

[eraf543-B167] Wong-Bajracharya J, Singan VR, Monti R, Plett KL, Ng V, Grigoriev IV, Martin FM, Anderson IC, Plett JM. 2022. The ectomycorrhizal fungus *Pisolithus microcarpus* encodes a microRNA involved in cross-kingdom gene silencing during symbiosis. Proceedings of the National Academy of Sciences, USA 119, e2103527119. 10.1073/pnas.2103527119 PMC8784151 35012977

[eraf543-B168] Wu H, Li B, Iwakawa H, et al 2020. Plant 22-nt siRNAs mediate translational repression and stress adaptation. Nature 581, 89–93. 32376953 10.1038/s41586-020-2231-y

[eraf543-B169] Wu Y, Wang S, Wang P, Nie W, Ahmad I, Sarris PF, Chen G, Zhu B. 2024. Suppression of host plant defense by bacterial small RNAs packaged in outer membrane vesicles. Plant Communications 5, 100817. 38217288 10.1016/j.xplc.2024.100817 PMC11009154

[eraf543-B170] Wytinck N, Ziegler DJ, Walker PL, Sullivan DS, Biggar KT, Khan D, Sakariyahu SK, Wilkins O, Whyard S, Belmonte MF. 2022. Host induced gene silencing of the *Sclerotinia sclerotiorum ABHYDROLASE-3* gene reduces disease severity in *Brassica napus*. PLoS One 17, e0261102. 36018839 10.1371/journal.pone.0261102 PMC9417021

[eraf543-B171] Xu M, Li G, Guo Y, et al 2022. A fungal microRNA-like RNA subverts host immunity and facilitates pathogen infection by silencing two host receptor-like kinase genes. New Phytologist 233, 2503–2519. 34981514 10.1111/nph.17945

[eraf543-B172] Yang Z, Wafula EK, Kim G, et al 2019. Convergent horizontal gene transfer and cross-talk of mobile nucleic acids in parasitic plants. Nature Plants 5, 991–1001. 31332314 10.1038/s41477-019-0458-0

[eraf543-B173] Yokoi A, Yoshioka Y, Yamamoto Y, et al 2017. Malignant extracellular vesicles carrying MMP1 mRNA facilitate peritoneal dissemination in ovarian cancer. Nature Communications 8, 14470. 10.1038/ncomms14470 PMC5343481 28262727

[eraf543-B174] Yu X, Lin X, Zhou T, Cao L, Kaixu H, Li F, Qu S. 2024. Host-induced gene silencing in wild apple germplasm *Malus hupehensis* confers resistance to the fungal pathogen *Botryosphaeria dothidea*. The Plant Journal 118, 1174–1193. 38430515 10.1111/tpj.16664

[eraf543-B175] Zand Karimi H, Baldrich P, Rutter BD, Borniego L, Zajt KK, Meyers BC, Innes RW. 2022. Arabidopsis apoplastic fluid contains sRNA– and circular RNA–protein complexes that are located outside extracellular vesicles. The Plant Cell 34, 1863–1881. 35171271 10.1093/plcell/koac043 PMC9048913

[eraf543-B176] Zand Karimi H, Innes RW. 2022. Molecular mechanisms underlying host-induced gene silencing. The Plant Cell 34, 3183–3199. 35666177 10.1093/plcell/koac165 PMC9421479

[eraf543-B177] Zanini S, Šečić E, Busche T, Galli M, Zheng Y, Kalinowski J, Kogel K-H. 2021. Comparative analysis of transcriptome and sRNAs expression patterns in the *Brachypodium distachyon*—*Magnaporthe oryzae* pathosystems. International Journal of Molecular Sciences 22, 650. 33440747 10.3390/ijms22020650 PMC7826919

[eraf543-B178] Zeng J, Gupta VK, Jiang Y, Yang B, Gong L, Zhu H. 2019. Cross-kingdom small RNAs among animals, plants and microbes. Cells 8, 371. 31018602 10.3390/cells8040371 PMC6523504

[eraf543-B179] Zhang L, Jing X, Chen W, et al 2019. Host plant-derived miRNAs potentially modulate the development of a cosmopolitan insect pest, *Plutella xylostella*. Biomolecules 9, 602. 31614786 10.3390/biom9100602 PMC6843310

[eraf543-B180] Zhang L, Wei Y, Wei L, Liu X, Liu N. 2023. Effects of transgenic cotton lines expressing *dsAgCYP6CY3-P1* on the growth and detoxification ability of *Aphis gossypii* glover. Pest Management Science 79, 481–488. 36196669 10.1002/ps.7220

[eraf543-B181] Zhang T, Zhao Y-L, Zhao J-H, Wang S, Jin Y, Chen Z-Q, Fang Y-Y, Hua C-L, Ding S-W, Guo H-S. 2016. Cotton plants export microRNAs to inhibit virulence gene expression in a fungal pathogen. Nature Plants 2, 16153. 27668926 10.1038/nplants.2016.153

[eraf543-B182] Zhang Z-L, Wang X-J, Lu J-B, et al 2024. Cross-kingdom RNA interference mediated by insect salivary microRNAs may suppress plant immunity. Proceedings of the National Academy of Sciences, USA 121, e2318783121. 10.1073/pnas.2318783121 PMC11032475 38588412

[eraf543-B183] Zhao J-H, Guo H-S. 2019. Trans-kingdom RNA interactions drive the evolutionary arms race between hosts and pathogens. Current Opinion in Genetics & Development 58–59, 62–69. 10.1016/j.gde.2019.07.019 31472442

[eraf543-B184] Zhu C, Liu J-H, Zhao J-H, Liu T, Chen Y-Y, Wang C-H, Zhang Z-H, Guo H-S, Duan C-G. 2022. A fungal effector suppresses the nuclear export of AGO1–miRNA complex to promote infection in plants. Proceedings of the National Academy of Sciences, USA 119, e2114583119. 10.1073/pnas.2114583119 PMC8944911 35290117

[eraf543-B185] Zhu K, Liu M, Fu Z, et al 2017. Plant microRNAs in larval food regulate honeybee caste development. PLoS Genetics 13, e1006946. 28859085 10.1371/journal.pgen.1006946 PMC5578494

[eraf543-B186] Zhu L, Zhu J, Liu Z, Wang Z, Zhou C, Wang H. 2017. Host-induced gene silencing of rice blast fungus *Magnaporthe oryzae* pathogenicity genes mediated by the brome mosaic virus. Genes 8, 241. 28954400 10.3390/genes8100241 PMC5664091

